# Engineering Cocrystals of Poorly Water-Soluble Drugs to Enhance Dissolution in Aqueous Medium

**DOI:** 10.3390/pharmaceutics10030108

**Published:** 2018-07-31

**Authors:** Indumathi Sathisaran, Sameer Vishvanath Dalvi

**Affiliations:** 1 Department of Biological Engineering, Indian Institute of Technology Gandhinagar, Palaj, Gujarat 382355, India; indumathi.s@iitgn.ac.in; 2 Department of Chemical Engineering, Indian Institute of Technology Gandhinagar, Palaj, Gujarat 382355, India

**Keywords:** crystal engineering, cocrystals, coformers, eutectics, polymorphism, poorly water-soluble, dissolution enhancement, hydrogen bonding

## Abstract

Biopharmaceutics Classification System (BCS) Class II and IV drugs suffer from poor aqueous solubility and hence low bioavailability. Most of these drugs are hydrophobic and cannot be developed into a pharmaceutical formulation due to their poor aqueous solubility. One of the ways to enhance the aqueous solubility of poorlywater-soluble drugs is to use the principles of crystal engineering to formulate cocrystals of these molecules with water-soluble molecules (which are generally called coformers). Many researchers have shown that the cocrystals significantly enhance the aqueous solubility of poorly water-soluble drugs. In this review, we present a consolidated account of reports available in the literature related to the cocrystallization of poorly water-soluble drugs. The current practice to formulate new drug cocrystals with enhanced solubility involves a lot of empiricism. Therefore, in this work, attempts have been made to understand a general framework involved in successful (and unsuccessful) cocrystallization events which can yield different solid forms such as cocrystals, cocrystal polymorphs, cocrystal hydrates/solvates, salts, coamorphous solids, eutectics and solid solutions. The rationale behind screening suitable coformers for cocrystallization has been explained based on the rules of five i.e., hydrogen bonding, halogen bonding (and in general non-covalent bonding), length of carbon chain, molecular recognition points and coformer aqueous solubility. Different techniques to screen coformers for effective cocrystallization and methods to synthesize cocrystals have been discussed. Recent advances in technologies for continuous and solvent-free production of cocrystals have also been discussed. Furthermore, mechanisms involved in solubilization of these solid forms and the parameters influencing dissolution and stability of specific solid forms have been discussed. Overall, this review provides a consolidated account of the rationale for design of cocrystals, past efforts, recent developments and future perspectives for cocrystallization research which will be extremely useful for researchers working in pharmaceutical formulation development.

## 1. Introduction

**Figure 1:**
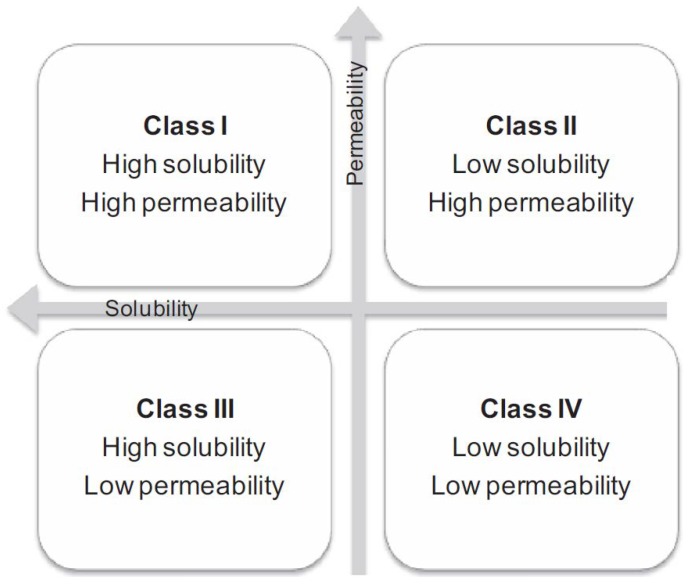
Schematic representation of Biopharmaceutics Classification System (BCS) of Drugs [Reprinted from [1] with permission from Elsevier].

Biopharmaceutics Classification System (BCS) of drugs classifies drugs into four major categories (Figure 1) based on their solubility and permeability behavior [1]. BCS Class II and Class IV drugs suffer from poor aqueous solubility. Poor aqueous solubility of hydrophobic drugs can result in poor absorption, low bioavailability and poses challenges for drug development process [2]. Enhancing bioavailability of poorly water-soluble BCS class II and BCS class IV drugs therefore becomes necessary to improve drug’s efficacy.

In addition to BCS of drugs, Developability Classification System (DCS) of drugs also plays a significant role in determining the development of pharmaceutical formulations, especially the oral formulations based on its solubility in biorelevant media such as FaSSIF (Fast State Simulated Intestinal Fluid) and FeSSIF (Fed State Simulated Intestinal Fluid) rather than its solubility in buffers [3,4,5]. Very few reports are available in the literature where researchers have determined the dissolution rate of cocrystals of poorly water-soluble drugs in biorelevant media [6,7]. The studies illustrate that cocrystals exhibited enhanced dissolution rate in biorelevant media and buffer as well indicating that the DCS serves as a highly relevant tool in determining developability of cocrystals of poorly water-soluble APIs.

Enhancing aqueous solubility of poorly water-soluble drugs without compromising on stability is one of the major challenges faced by the pharmaceutical industries during drug discovery and development processes [8,9,10,11,12]. Crystal Engineering is a tool which can be used to tailor the physicochemical properties of Active Pharmaceutical Ingredients (APIs) [13] such as melting point, dissolution rate, aqueous solubility, refractive index, surface activity, habit, density, electrostatic, mechanical and optical properties [14]. Cocrystallization is one of the crystal engineering approaches adopted to prepare multicomponent pharmaceutical crystals to enhance the dissolution rates of poorly water-soluble APIs without affecting their intrinsic properties [15].

Tremendous research is now being conducted in cocrystallization. Discovery of new cocrystals is now considered as one of the patentable invention [16]. Cocrystal technology is being considered as an advanced technology used for improving the drug product by modifying the molecular conformations and intermolecular interactions [17]. Combination drug therapy (combining two drugs in a solid form and administering in a single dose) is considered to be another important advantage of the cocrystal technology [18,19]. However, the number of marketed cocrystal products available till date is very low [20,21]. Examples of such cocrystals available in market and approved by the Food and Drug Administration (FDA) are Entresto, Lexapro [20] and Depakote [21].

In this review, we provide a consolidated account of information available in the literature on cocrystal engineering. We have discussed different aspects of cocrystallization technology such as the synthesis techniques, type of intermolecular interactions involved in cocrystallization, different types of solids obtained during cocrystallization, techniques to characterize cocrystals, different types of cocrystals and dissolution of cocrystals in aqueous medium.

## 2. Types of Solid Forms Obtained from Cocrystallization

**Figure 2:**
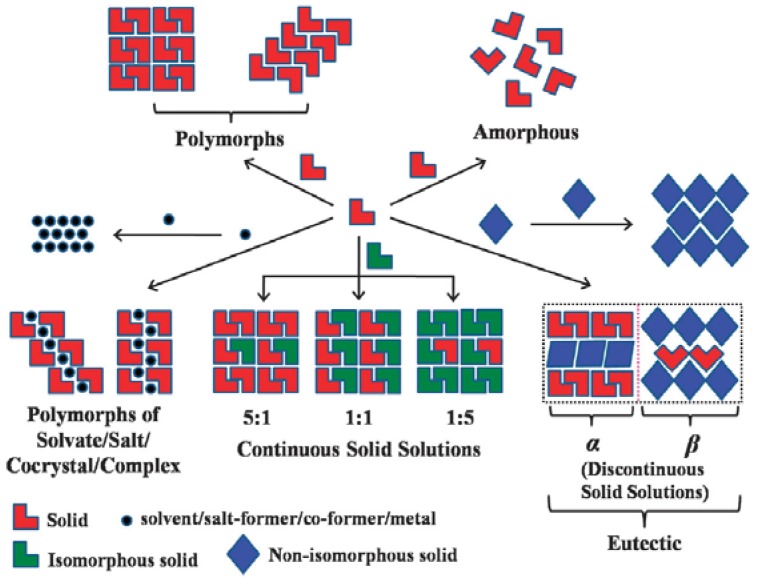
Schematic representation of various single and multicomponent forms of an API [Reprinted from [34] with permission. Copyright 2014 Royal Society of Chemistry].

While cocrystal formation is the main desired outcome, an unsuccessful cocrystallization event might result in formation of different solid forms such as eutectics [22,23,24,25,26], a drug polymorph [27,28], coamorphous solids [25,28,29,30,31,32,33], physical mixtures [22,25,34], salts [35], solvates [35,36], hydrates [35,36,37] and solid solutions [38,39,40]. These solid forms can consist of a single component or multiple components. Single component solids include amorphous forms and polymorphs. Multicomponent solids can be cocrystals [41,42], salts [41], coamorphous solids [28], polymorphs of cocrystals [43], solvates/hydrates [36,37], and continuous solid solutions or discontinuous solid solutions/eutectics [34]. Figure 2 and Table 1 explains the differences between different solid forms of an API.

**Figure 3:**
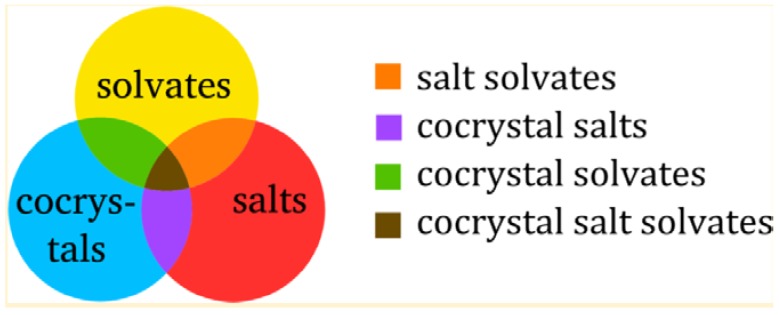
Schematic representation of proposed multicomponent classification system by Grothe et al. [46] [Reprinted from [46] with permission. Copyright 2016 American Chemical Society].

As new forms of pharmaceutical solids are being produced with different type of guest molecules (solvents/water/solids), assigning an exact nomenclature for each of the solid was a hotly debated topic for many years [44,45,46]. Inspired by the work of Aitipamula et al. [45], Grothe et al. [46] developed a straightforward system for classification of multicomponent solid forms. The classification system proposed by Grothe et al. [46] is shown in Figure 3.

### 2.1. Cocrystals

Cocrystals are defined as crystalline materials with two or more different molecules (i.e., drug and coformers) in the same crystal lattice [47]. In pharmaceutical industries, cocrystals have gained a tremendous importance because of its ability to fine-tune the physicochemical properties of drugs [48]. Regulatory agencies such as United States Food and Drug Administration (USFDA) and European Medicine Agency (EMA) has provided distinct definition for these pharmaceutical cocrystals [49,50]. USFDA defines cocrystals as ‘*crystalline materials composed of two or more molecules in the same crystal lattice’* [49]. According to EMA, cocrystals are *‘homogenous (single phase) crystalline structures made up of two or more components in a definite stoichiometric ratio where the arrangement in the crystal lattice is not based on ionic bonds (as with salts)’* [50]. Cocrystals are mainly stabilized by the strong intermolecular non-covalent adhesive interactions of short-range order [51] that exist between the drug and coformer molecules. The first known cocrystal called ‘quinhydrone’ was synthesized using benzoquinone and hydroquinone by Friedrich Wohler in the year 1844 [52]. It was the first cocrystal structure reported in Cambridge Structural Database [53]. Intermolecular interactions [54], structural compatibility [55] and stoichiometry of API and coformer molecules [56] determine successful formation of cocrystal during a cocrystallization event. Table 2 presents the summary of a few literature reports available on representative pharmaceutical cocrystals.

### 2.2. Eutectics

Eutectics are another important class of multicomponent solids that has been gaining tremendous attention in pharmaceutical research in the recent years. When attempts to obtain a cocrystal fail, one may end up with a eutectic. Eutectics are multicomponent crystalline materials which exist in the form of discontinuous solid solutions (as shown in Figure 2). Cherukuvada and Nangia [34] defined eutectics as a conglomerate of solid solutions [34]. Unlike cocrystals, eutectic phases are stabilized by weaker adhesive interactions between the unlike molecules or stronger cohesive interactions between the molecules having similar structure [34,55]. Eutectic phases do not have a distinct or a specific crystal structure. Instead their crystalline nature resembles the combination of crystalline nature of the parent components. Eutectics possess a melting point less than the melting point of the pure components. Table 1 presents characteristic features of eutectics vis-à-vis’ other solid forms.

Eutectics can be as good as cocrystals in fine-tuning the physicochemical properties of an API [55]. Duarte et al. [76] reported a eutectic dispersion of fenofibrate with a low molecular weight polymer, polyethylene glycol. This eutectic dispersion is commercially being available with the trade name ‘Fenoglide’ [76,77]. These eutectic dispersions possessed lower melting point and crystalline nature similar to the parent components [76]. Furthermore, Faeges [78] formulated liquefied form of a eutectic of aspirin with 2-3 parts of glycerin or propylene glycol (*w/v*) [78] as an ointment for topical applications. These eutectic formulations were also reported to enhance the shelf-life of aspirin by preventing its hydrolysis [78].

Several reports are available in the literature where eutectics were used for increasing the dissolution of poorly water-soluble drugs. The summary of reports available in the literature on various drug eutectics and their dissolution behavior are presented in Table 3. From Table 3, it is evident that the eutectic mixtures show significantly enhanced dissolution as compared to the raw drug. This ability of eutectic mixtures to exhibit enhanced dissolution rates than the raw drug is attributed to the randomized lattice arrangement which exists in the eutectic phase [24].

Cherukuvada and Row [55] employed hydrogen-bonding principles to understand and design generalized rules involved in cocrystal/eutectic/solid solution formation by investigating cocrystallization of 4,4′-bipyridine, isonicotinamide, isoniazid Fluoxetine hydrochloride drug systems as a model for their study [55]. Furthermore, Cherukuvada and Nangia [34] formulated ground rules based on the crystal engineering approach to explain the circumstances favoring cocrystal/eutectic/solid solution formation with respect to the structural similarity and intermolecular interactions existing in the binary system. While eutectic formation occurs with a pair of molecules having either similar or dissimilar structures [23,34] and sustained by weaker adhesive interactions, cocrystals are stabilized by strong adhesive interactions [34]. Table 1 presents the characteristic features of cocrystals vis-à-vis eutectics and solid solutions in comparison with cocrystals.

### 2.3. Solid Solutions

A solid solution is a homogeneous phase formed out of a solid-state reaction between the drug and coformer which are miscible into each other. When two molecules possess structural similarity (isomorphous and isostructural), then the resultant solid form is generally a solid solution [38,39,40]. Solid solutions are stabilized by strong cohesive interactions. Table 1 presents salient features of solid solutions and explains the difference in how different a solid solution is vis-à-vis other solid forms. Table 4 presents the summary of a few reports available in the literature on drug solid solutions.

### 2.4. Coamorphous Solids

Coamorphous solids are another interesting group of pharmaceutical solids. Coamorphous solids are solid forms in which the amorphous state is stabilized by weak intermolecular interactions [85,86] between the drug and coformer molecules. Coamorphous solids are used to enhance the bioavailability of hydrophobic drugs as their amorphous nature can improve aqueous solubility of an API. The term ‘coamorphous’ was first coined by Chieng et al. [85]. A drug and the polymer combination may result into ‘Amorphous Solid Dispersions (ASDs)’ whereas a mixture of amorphous drug and low molecular weight coformers can form a ‘coamorphous solid’ [87]. Weak intermolecular interactions (such as hydrogen bonding and/or Π-Π interactions [85,86]) dominate between drug and coformer in coamorphous solids. The coamorphous solids possess glass transition temperature (T_g_) in between the glass transition temperatures of individual components [86]. There are also reports where coamorphous solids with no well-defined stoichiometry have been reported [30]. Table 5 presents a summary of few literature reports where coamorphous solids of APIs have been reported.

From the literature reports (as shown in Table 5), it is evident that the coamorphous solids showed enhanced dissolution rates than the raw drug molecules [25,28,29,30,31,33,88]. This enhancement in dissolution rate mainly depends on two important factors namely: (i) capability of the drug to maintain higher supersaturation level and (ii) the strength of the API-coformer interaction to slow down the nucleation. In a study on Curcumin-artemisin coamorphous solid, Nangia and coworkers [28] have stated that coamorphization induces micronization of particles and in a way also leads to enhanced dissolution rates [28]. From the literature information available on coamorphous solids, one can conclude that coamorphous solids are one of the byproducts of a cocrystallization process which can enhance dissolution of hydrophobic drugs in aqueous medium [89], and in turn their bioavailability. Also, it was observed that the higher level of apparent solubility attained with the coamorphous solids during in vitro dissolution studies also resulted in enhanced in vivo bioavailability [89,90]. However, there is a need to investigate the long-term dissolution rates of pharmaceutical coamorphous solids.

### 2.5. Salts

**Figure 4:**
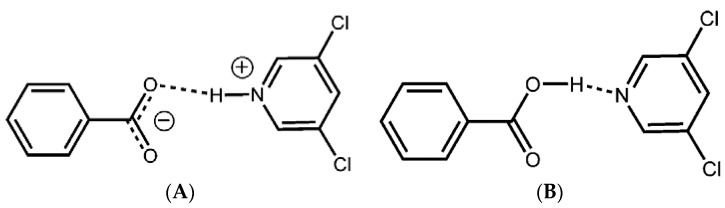
Schematic representation of difference between (**A**) a salt and (**B**) a cocrystal [Reprinted from [91] with permission. Copyright 2007 American Chemical Society].

Salt formation is one of the traditional methods employed to enhance the aqueous solubility of poorly water-soluble drugs. More than half of the medicines in market exist in the form of salt [90]. Salts are formed because of intermolecular hydrogen bonding due to proton transfer between the molecules with ionizable functional groups. Figure 4 presents the schematic representation of the difference between a salt and cocrystal [91]. Hence, salt formation is favored only when the API contains an ionizable site in it [15,92]. Formation of an API salt with a coformer occurs when there is a proton transfer from an acid to a base in the ionic state [91,93]. According to Sarma et al. [44], API molecules with nitrogenous functionality and –COOH functional groups in their chemical structure are more susceptible to salt formation [44] since these molecules can favor proton transfer to the coformer molecules. It has been also reported that presence of adequate number of counterions in an API and coformer molecule facilitates salt formation [94]. The presence of charge-assisted hydrogen bonding in salts enables coformer molecules to dissociate easily from the salt complexes resulting in peak solubility of drug in a dissolution medium within a few hours. Moreover, from thermodynamic point of view, salts possess higher enthalpy of hydration which also facilitates attainment of higher dissolution rate [95].

### 2.6. Salt-Cocrystal Continuum

Salt-cocrystal continuum is other interesting subset of multicomponent pharmaceutical solids which falls under cocrystal/salt category. When a multicomponent solid form contains mixed ionization states (the extent of proton transfer from one molecule to the other is not predictable), it is difficult to understand whether the resultant solid form is a salt or cocrystal. This mainly occurs when the difference in pKa value of the drug and coformer lies between 0 and 3 [93] (Explained later in Section 3.1). Childs et al. [93] investigated the influence of crystal structure on the ionization states of the salt-cocrystal continuum. Jacobs and Noa [96] reported a hybrid salt-cocrystal methanol water solvate of p-Coumaric acid-Quinine with unexpected stoichiometry prepared by slow evaporation [96].

## 3. Factors Determining Cocrystallization

### 3.1. ∆pKa Rule

∆pKa value has been used to assess cocrystal formation ability of a coformer with a given API [93,97]. pKa value (negative logarithm of dissociation constant) indicates the ability of an acid molecule to give up a proton [93]. When the difference between pKa value of API and coformer (∆pKa) ranges in negative values, there will be no proton transfer [93,98]. Therefore, one can possibly expect cocrystal formation in such a case [93,97,98,99]. On the other hand, salt formation is observed when ∆pKa value is greater than 3 due to completion of proton transfer [93,98,99]. According to Berry and Steed [98], when ∆pKa value remains close to that of a base, then the system forms a salt and when it exists close to the acid, then the system forms a cocrystal [98]. da Silva et al. [71] designed and developed five 5-Fluorocytosine cocrystals with adipic, succinic, terephthalic, benzoic, and malic acid based on the ∆pKa values for API-coformer [71]. When the ∆pKa value ranges between 0 and 3 (partially ionized states), in such cases, these solid forms were referred to as Salt-cocrystal continuum [93,99,100]. Interestingly, Nangia and coworkers [100], while attempting to develop Clotrimazole (CLT) cocrystals with some carboxylic acid coformers, identified salt formation with maleic acid (MA) at a stoichiometric ratio of 1:0.5 (CLT:MA) whereas the calculated ∆pKa value for the system was 0.93 [101]. Thus, an ∆pKa value cannot always be used to predict/confirm the nature of a solid phase in all the cases and an experimental analysis is required for accuracy.

### 3.2. Hydrogen Bond Donors and Acceptors

The number of hydrogen bond donors and acceptors in a coformer and drug molecules also determines the extent of success in a cocrystallization event. Molecules that can form multiple hydrogen bonds are likely to form cocrystals with the coformer molecules [102]. Etter [103] and Donohue [104] framed HydrogenBond Rules to predict the circumstances under which hydrogen bond interactions that result into cocrystals [54,103,104]. These rules are as given below:

- Mostly all good proton donors (such as –COOH, –NH_4_^+^) and acceptors (such as –OH, –NH_3_) are utilized in hydrogen bonding.
- Six-membered ring intramolecular hydrogen bonds (such as C-H…O) are formed first in preference to intermolecular hydrogen bonds (such as N-H…O and O-H…O)
- The best proton donors and acceptors available after intramolecular hydrogenbond formation then participate in intermolecular hydrogen bonds
- All acidic hydrogen atoms are included in hydrogen bonding in the crystal structure

### 3.3. Molecular Recognition Points

Almarsson and Zaworotko [105] pointed out that the API molecules contain certain functional group (or molecular recognition point) in their structure which interacts with the coformer and thereby create a supramolecular unit (or molecular recognition point) called supramolecular synthons [105]. The term ‘Synthon’ was first introduced by Corey in 1967 who defined Synthons as “*Structural units within supermolecules which can be formed and/or assembled by known or conceivable synthetic operations involving intermolecular interactions*” [106]. Desiraju [107] defined supramolecular synthons as spatial arrangement of intermolecular interactions which serves as a base for any supramolecular synthesis [107]. Thus, synthons are design elements in crystal engineering which are different from the term ‘intermolecular interactions’ [108]. Sometimes, a synthon can also be represented as a single interaction [107] (such as Cl…Cl and N…Br interactions) in a few supermolecular structures.

**Figure 5:**
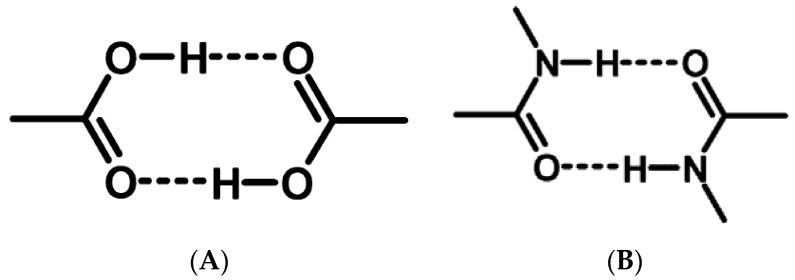
Schematic representation of supramolecular homosynthons (**A**) acid-acid dimer and (**B**) amide-amide dimer [34,55] [Reprinted from [55] with permission. Copyright 2014 American Chemical Society].

**Figure 6:**
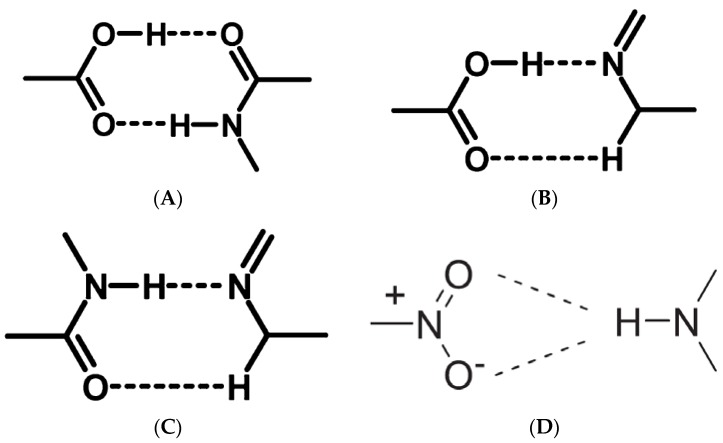
Schematic representation of supramolecular heterosynthons (**A**) acid-amide dimer [29,50], (**B**) acid-pyridine dimer [34,55], (**C**) amide-pyridine dimer [34,55] [Reprinted from [55] with permission. Copyright 2014 American Chemical Society] and (**D**) Nitro-amine interaction [98] [Reprinted from [98] with permission from Elsevier].

**Figure 7:**
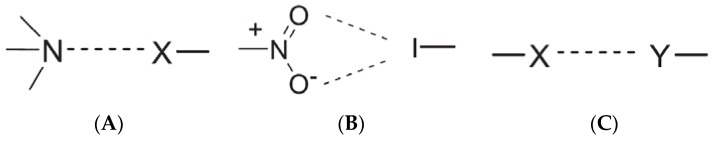
Schematic representation of supramolecular heterosynthons brought about by (**A**) Amine-halogen interaction, (**B**) Nitro-iodo interaction and (**C**) Halogen bonding (X-Halogens such as Cl, Br, I and Y-Electron pair donors such as N or O) [98] [Reprinted from [98] with permission from Elsevier].

Based on the complementary functional groups in the drug and coformer, these supramolecular synthons are classified as homosynthons and heterosynthons [105]. Homosynthons are formed as a result of the interaction between self-complementary functional groups such as acid…acid and amide…amide groups (Figure 5) whereas the heterosynthon formation arises due to the interaction between two different functional groups (such as acid…amide, acid…pyridine and amide…pyridine groups (see Figure 6). Heterosynthons could also be formed as a result of halogen bonding. Figure 7 presents a few examples of heterosynthons formed through halogen bonding.

### 3.4. Flexibility of Synthon-Forming Functional Groups

In addition to molecular recognition points, the position of functional groups and the conformational flexibility of participating molecules play a significant role in determining success rate of a cocrystallization. For instance, Nangia and coworkers [22,72] identified that resorcinol can cocrystallize with curcumin whereas hydroquinone and catechol could not cocrystallize with curcumin though all the three molecules possessed same functional groups [22,72]. Such observations suggest that understanding the rationale behind formation of supramolecular synthons in the crystal lattice of the cocrystals is highly necessary for cocrystal design and development.

Aakeroy et al. [108] carried out an extensive study to understand how polymorphic compounds serve as good cocrystallizing agents/coformers and emphasized the significance of flexibility of synthon-forming functional groups of coformers during any cocrystallization [108]. They experimentally studied the cocrystal forming ability of three polymorphic compounds, isonicotinamide, 2-amino 3-nitropyridine, 4-chlorobenzamide and maleic hydrazide [108]. It was observed in their study that isonicotinamide, 2-amino 3-nitropyridine and 4-chlorobenzamide participated actively in intermolecular hydrogen bonding with a variety of aliphatic and aromatic carboxylic acids and thereby favored the formation of binary/ternary cocrystals whereas maleic hydrazide was not found suitable candidate for cocrystallization with aromatic or aliphatic compounds having acid, amide and oxime functional groups. This was attributed to the flexibility of functional groups in coformers in addition to their polymorphic nature. Isonicotinamide, 2-amino 3-nitropyridine and 4-chlorobenzamide exhibit hydrogen bonding between different functional groups in each of their polymorphs whereas all the three polymorphs of maleic hydrazide always exhibited the primary hydrogen-bonding interactions between same functional groups which decreases the possibility of formation of new hydrogen bond synthons with the other molecules.

### 3.5. Carbon Chain Length of Dicarboxylic Acid Coformers

Carboxylic acids are some of the most commonly used coformers for cocrystallization of many small molecules since they can form heterosynthons with molecules containing amide and pyridine functional groups and homosynthons with API molecules containing acid functional group. However, the cocrystal forming tendency of carboxylic acids also depends on the length of carbon chain in it. Shevchenko et al. [109] while investigating cocrystallization of itraconazole with different aliphatic dicarboxylic acids containing carbon chains of varying length observed that as the length of carbon chain in the coformer molecules increases, the packing of these molecules within the crystal lattice of drug molecules becomes increasingly difficult due to steric hindrance or incompatibility of large carbon chain to exactly fit into the crystal lattice of drug [109]. During their study, the researchers identified that itraconazole formed cocrystals with oxalic acid (C2), adipic acid (C6), malonic acid (C3), glutaric acid (C5) and pimelic acid (C7) and not with suberic acid (C8), azelaic acid (C9) and sebacic acid (C10). Based on this observation, it can be safely concluded that coformers containing longer carbon chains possibly are not suitable candidates for cocrystallization with API molecules where the probability of geometrical fitness of coformer into the API lattice is low [109].

### 3.6. Effect of Solvents

Solubility of the API and coformer in a solvent used for cocrystallization plays a significant role in determining the success of cocrystallization experiment. The solubility of the individual components must be determined a priori to cocrystallization experiments [110]. The Phase Solubility Diagram (PSD), also called as Ternary (API-Coformer-Solvent) Phase Diagram can be constructed using this data which then serves as a fundamental tool to identify region of cocrystal formation, understand the solution chemistry and solubility behavior of cocrystals [110]. The polarity of the solvent system, solubility of API and coformer, temperature and pH are the important parameters which determine the cocrystal forming zone in a ternary system. Robertson and his coworkers [111] observed that the polarity of the solvent determined the type of non-covalent interactions (hydrogen-bond or halogen-bond), and thereby controlling intermolecular interactions in the cocrystal phases [111]. It was summarized that the hydrogen-bonded cocrystals formation was favored by less polar solvents (such as toluene) whereas the more polar solvents (such as chloroform, dichloromethane, acetone, acetonitrile, nitromethane and 1-propanol) favored the formation of halogen-bonded cocrystals and in some cases, mixed halogen and hydrogen-bonded cocrystals [111]. This is mainly attributed to the influence of polarity of the different solvents on the strength of intermolecular interactions.

## 4. Screening Methods for Cocrystals

As cocrystallization is influenced widely by several important parameters, selecting a suitable coformer for cocrystallization requires an effective screening process. Screening of a suitable coformer can be carried out experimentally or computationally. Experimental methods are exhaustive and time-consuming. On the other hand, computational methods can serve as a rapidscreening tool for initial assessment of coformers that are suitable for cocrystallization process. Section 4.1 and Section 4.2 presents the various computational and experimental methods that can be used for coformer screening.

### 4.1. Computational Methods

Most of the computational methods reported for coformer screening in literature till date are mainly thermodynamics-based methods. Issa et al. [112] used lattice energy calculations as an effective approach to screen coformers to form thermodynamically stable cocrystals [112]. If the lattice energy of the cocrystal is lower than the sum of lattice energies of individual components, then the cocrystal phase is said to be a thermodynamically stable phase [112]. Apart from calculation of lattice energies [112,113,114,115], several other parameters have also been employed for computational prediction of successful cocrystal formation or coformer screening. These include calculation of interaction energies [116,117], electrostatic potentials [118], molecular complementarity between API and coformers [119,120], solubility behavior [121,122,123], crystal energy landscapes of API-coformer pairs [124] and hydrogen bond propensities [125]. Table 6 presents the summary of various computational coformer screening methods [112,113,114,115,116,117,118,119,120,121,122,123,124,125,126,127,128,129,130,131,132] reported so far in the literature. Despite being less labor intensive, computational screening methods suffer from the requirement of large simulation times to perform molecular dynamic simulations.

### 4.2. Experimental Methods

Newman [133] has summarized various methods reported in the literature to obtain various solid forms such as polymorphs, salts, cocrystals and amorphous solid dispersions [133]. Though hydrogen-bonding motif structures play a significant role in stabilizing the crystal structures of cocrystals [134,135,136,137,138,139], in some cases, evaluation of crystal structures of some cocrystals synthesized using techniques such as Solution crystallization, mechanocrystallization and hot-stage microscopy showed that these cocrystals do not possess hydrogen-bonding motifs as given by Crystal Engineering principles [124]. Therefore, a suitable coformer screening technique is highly essential for a cocrystallization process. Given below is a brief account of different experimental techniques which could be used for screening coformers for cocrystallization.

#### 4.2.1. Differential Scanning Calorimetric (DSC) Analysis

Thermal analysis can be used for selection of a suitable coformer to form a cocrystal with a desired drug molecule. While one can detect the polymorphic transformation exhibited by the drug/coformer, a formation of a new phase (such as cocrystal), with the multicomponent reactant molecules (API and coformer) can also be easily detected. DSC has been reported to be a rapid thermal screening tool by Lu et al. [62]. Similarly, Yamashita et al. [140,141] conducted an extensive study using DSC as a coformer screening tool to identify formation of many pharmaceutical cocrystals [140,141]. Recently, Saganowska and Wesolowski [142] used DSC as a rapid screening tool and identified 15 different benzodiazepine cocrystals (at stoichiometric ratio of 1:1) [142].

DSC can be used as a tool to identify the coformers capable of forming a cocrystal with an API molecule using either of the following two approaches:

**(a) Nature of DSC Thermograms**

The nature of a DSC thermogram obtained for a binary mixture of API and coformer at a specific stoichiometric ratio can help in identifying the nature of the solid form as explained below:

**Physical mixture**—If heating of a binary mixture at a specific heating rate yields two endotherms, each corresponding to the melting point of the individual components, in a DSC thermogram, then it suggests that the binary mixture remains as a physical mixture with no intermolecular interactions occurring between these molecules [25,34].

**Eutectic**—A eutectic is said to have formed when the DSC thermogram shows a single endotherm with melting temperature less than the melting points of either of the parent molecules (the drug and the coformer) [34,55,62,140,141].

**Cocrystal**—A binary system can be said to be capable of forming a cocrystal when the DSC thermogram exhibits a single endotherm with melting points lower or in between or greater than the melting points of the individual components [15,34,140,141]. DSC analysis along with single crystal XRD analysis can confirm the formation of a new cocrystal phase.

Furthermore, if a DSC thermogram shows consecutive multiple peaks, it possibly indicates the cocrystal formation. In case of presence of two consecutive peaks, the first melting peak corresponds to the eutectic melting, followed by the cocrystal melting (represented by the second endotherm). It is also possible that two or multiple peaks are an indication of polymorphic transformation of cocrystals [62,140].

In case of presence of three consecutive endotherms in a DSC thermogram, the first represents the eutectic melt, the second represents the melting of excess component (drug or coformer), followed by the final melting of cocrystal in the third endotherm [24,62,140].

**Coamorphous solid**—The DSC thermogram of coamorphous solids show a glasstransition phase change implying the formation of a coamorphous solid phase [25,28,143].

**Solid solution**—If a DSC thermogram exhibits a simple solidus-liquidus behavior, it indicates the formation of a solid solution [34]. When two components in a binary system form a complete solution in both the solid and liquid phases, then this behavior is termed a simple solidus-liquidus behavior. If the two components in the binary system possess similar crystal structure, then the intermolecular interactions in these compounds remains similar and the difference in the size of atoms remains small. Such isomorphous compounds can result into a solid solution.

**(b) Binary Phase Diagrams**

DSC thermograms obtained for different mixtures containing drug and coformer molecules in varying stoichiometric ratios can be used to construct a binary phase diagram for any drug-coformer pair. These binary phase diagrams can be used to detect the cocrystal formation zone for a given system. Section 4.2.2 explains this in detail.

#### 4.2.2. Phase Diagrams

Phase diagrams are utilized as means to identifying different solid phases that can be formed between any drug-coformer pair. These phase diagrams can be generated either for two components (API-coformer) or for three components (API-coformer-solvent) as well.

**(a) Binary Phase Diagrams**

Binary phase diagrams are generally constructed with the data points obtained from thermal analysis methods such as DSC analysis [140]. The onset temperature of the first endotherm in a DSC thermogram is generally chosen as solidus point. The peak temperature of the second endotherm is chosen as liquidus point for constructing the phase diagram. The melting behavior of an API and coformer (congruent/incongruent melting) determines the solid solution/eutectic and cocrystal forming property for the investigated system. In general, the eutectic forming binary system adopts ‘V’-shaped curve whereas the cocrystal forming system adopts ‘W’-shaped curve indicating cocrystal formation between two eutectics. Reports are available in the literature where researchers have used binary phase diagram for determining the cocrystal formation zone for the investigated drug-coformer combination [24,25,34,50,73,140,141,144,145]. Sangster [80] has reported a set of binary phase diagrams for 60 different drug systems constructed with the help of computer-coupled phase diagram/thermodynamic analysis [80].

**Figure 8:**
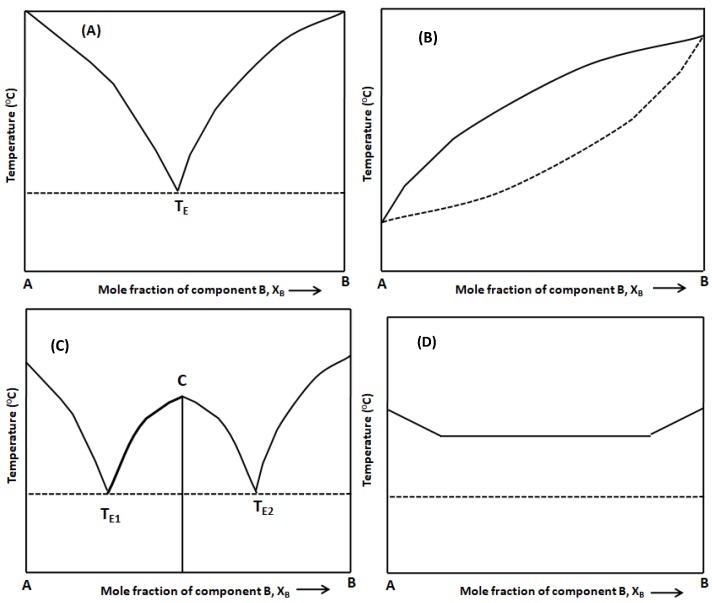
Schematic representation of Binary phase diagrams for (**A**) Eutectic formation [A and B—Binary components; T_E_—Eutectic temperature] [24,55,79,140], (**B**) Formation of solid solution [34], (**C**) Cocrystal formation [A and B—Binary components; C—Cocrystal phase; T_E1_ and T_E2_—Eutectic temperatures] [24,73,140,145] and (**D**) Physical mixture [Dotted line-Onset of first component melting and dark line-Melting of the second component] [25].

The commonly observed binary phase diagram for most of the drug-coformer pairs reported so far in the literature are shown in Figure 8.

**(b) Ternary Phase Diagrams**

**Figure 9:**
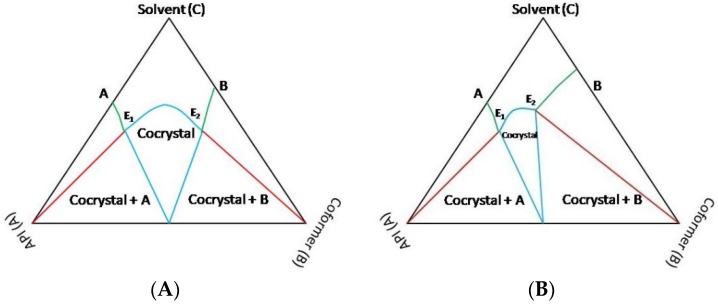
(**A**) Schematic representation of (**A**) a symmetric ternary phase diagram and (**B**) an asymmetric ternary phase diagram [A—API; B—Coformer, C—Solvent, E_1_ and E_2_—Eutectic points] [148].

Solution crystallization experiments can result in cocrystals. However, sometimes multicomponent single crystals are not formed during solution crystallization due to the incongruent solubility behavior of individual components in the solvent system. Therefore, the thermodynamic behavior of a system involving ternary components needs to be ascertained before attempting solution crystallization [146]. Ternary Phase Diagrams (Solute-solute-solvent phase diagram) help in determining the cocrystal formation region for a given system. Many researchers have employed ternary phase diagram as an optimization tool to determine suitable stoichiometric ratio of API and coformer for cocrystal formation [146,147,148,149,150]. When the two components manifest a similar/congruent solubility, the phase diagram exhibits a more symmetrical trend [148]. On the other hand, when the two components show dissimilar/incongruent solubility, then the phase diagram will be less symmetrical behavior [148]. Figure 9A,B presents schematics of a symmetric and an asymmetric ternary phase diagram.

## 5. Synthesis of Cocrystals

Cocrystals can be synthesized using several methods which could either be the batch processes or continuous processes. Given below is a brief account of different techniques which are being used to synthesize cocrystals.

### 5.1. Batch Processes

The batch mode cocrystallization techniques include mechanochemical methods such as Solid-State grinding [151,152], Liquid-Assisted grinding [151], Ion and Liquid-Assisted Grinding [153] and solvent-based techniques such as slow evaporation [154], ultrasound-assisted cocrystallization [155,156], slurry conversion [140,157] or solvent-mediated phase transformation [158], generation of cocrystals from moisture [159,160] and anti-solvent precipitation (Liquid Anti-Solvent precipitation) [161] and Gas Anti-Solvent precipitation [162]. Table 7 presents the principle involved behind these batch cocrystallization techniques [151,153,154,155,159,161,162,163]. Mechanochemical methods, ultrasound-assisted cocrystallization, cocrystal generation using moisture are discussed in the Section 5.1 in detail.

**(a) Mechanochemical Methods (Grinding)**

The API and coformer molecules can be cocrystallized by grinding together without solvent, which is termed as Solid-State Grinding (SSG) or Neat grinding or with a few drops of solvent [termed as Solvent-Drop grinding or Liquid-Assisted Grinding (LAG)]. During grinding, the low molecular weight components (solid/liquid/gas phase) diffuse easily into the API crystal lattice [152] which can result in a formation of intermediate phases such as eutectic or amorphous phase which can further result in formation of a cocrystal. According to Etter [103], conversion of individual parent molecules into hydrogen-bonded cocrystals basically occurs based on the rate and force of grinding, particle size of the components and vapor pressure of the phases [103].

Chadwick et al. [152] explained different mechanisms of cocrystal formation via grinding and the various thermodynamic parameters that influence cocrystal formation during grinding. Friscic [153] explored ion and liquid-assisted grinding (ILAG) in designing multicomponent cocrystals. It was proposed that grinding of multiple components along with liquid facilitates diffusion of liquid phase into solid-state thereby enabling mobility of molecules and exposing the hidden molecules to the surface.

Numerous reports are available in the literature where grinding (solid-state or liquid-assisted) methods were used extensively to produce cocrystals [164,165,166,167,168,169,170,171,172]. Table 8 presents a summary of a few reports available in the literature where the researchers have proposed scientific mechanisms behind solid-state or liquid-assisted grinding process [151,152,167,168,171,173,174].

**(b) Ultrasound-assisted Cocrystallization**

Ultrasound has been widely used for inducing nucleation in solution and cocrystallizing small molecules. Ultrasound-assisted cocrystallization has several advantages over conventional solution crystallization. The mechanical energy released during passage of ultrasonic waves induces primary nucleation at lower supersaturation levels, thereby reducing the induction time and metastable zone width [175,176]. Ultrasound can therefore induce crystallization easily from solution which otherwise is difficult to attain by conventional solution crystallization experiments [155]. Reports are available in the literature where ultrasound-assisted solution cocrystallization (USSC) [60,155,156,177] has been reported as a method to crystallize caffeine-maleic acid (2:1) [155] and caffeine-oxalic acid (2:1) [155,156].

Morrison et al. [178] proposed a bench-top inexpensive method called ‘Solvent-Drop Floating Foam Rack/Sonic Bath Method’ for synthesizing cocrystals and tested the feasibility of this method for synthesis of carbamazepine-saccharin (1:1) form II, carbamazepine-nicotinamide (1:1), AMG 517-benzoic acid (1:1) and AMG 517-malic acid (1:1) cocrystals. In this technique, the moist powders were subjected to sonication in a sonic bath for an optimized period (90 min was used for carbamazepine and AMG 517 drug systems since sonication for less than 5 min yielded only partial conversion of cocrystal for many systems) using a floating foam rack for inducing cocrystallization [178]. The time duration provided for sonication and a suitable solvent selection were found to be the two critical parameters which influence cocrystal formation by this technique [178].

**(c) Cocrystal Generation Using Moisture**

**Figure 10:**
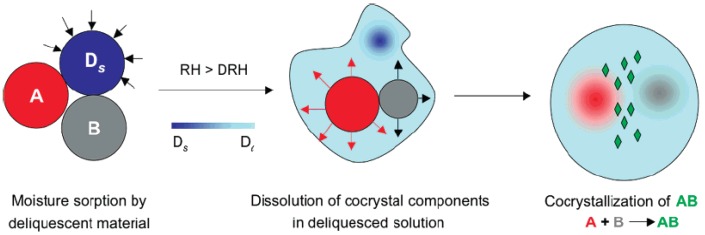
Schematic representation of cocrystal formation by moisture uptake in presence of a deliquescent additive [Reprinted from [159] with permission. Copyright 2007 American Chemical Society].

Rodriguez-Hornedo and coworkers [159] conducted a study with API molecules namely carbamazepine, theophylline and caffeine to understand the mechanism behind cocrystal formation in presence of deliquescent additive such as sucrose or fructose [159]. Figure 10 presents a schematic of cocrystal formation from moisture in presence of deliquescent additive. The study concluded that uptake of moisture by the solid reactants facilitated nucleation and growth of carbamazepine-nicotinamide, carbamazepine-saccharin, caffeine-maleic acid, caffeine-oxalic acid, caffeine-malonic acid, caffeine-glutaric acid, theophylline-maleic acid, theophylline-oxalic acid, theophylline-malonic acid and theophylline-glutaric acid cocrystals.

**Figure 11:**
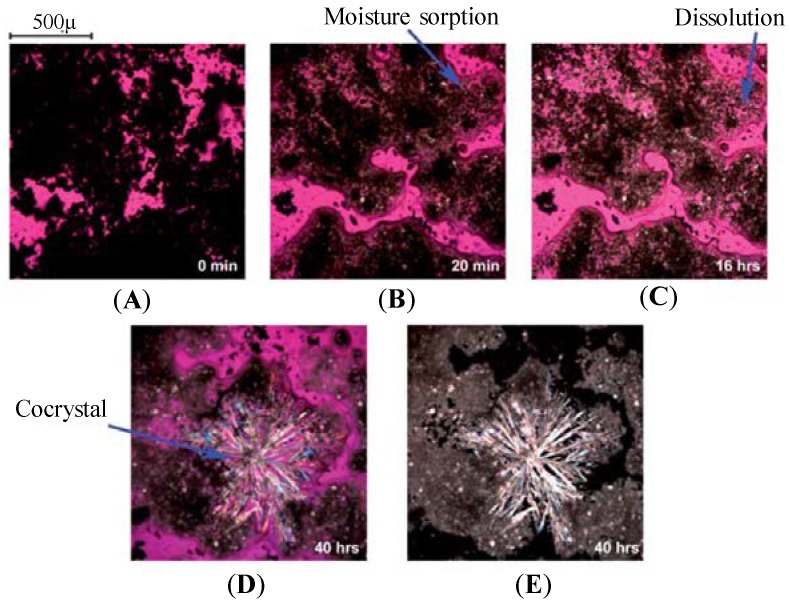
Optical microscopic images presenting (**A**–**C**) Dissolution of CBZ and NCT and (**D**,**E**) CBZ-NCT cocrystal formation by moisture sorption by PVP K12 in equimolar concentration of CBZ and NCT at 75% RH and 25 °C [Reprinted from [160] with permission. Copyright 2011 Royal Society of Chemistry].

Furthermore, Good et al. [160] attempted to understand the mechanism behind carbamazepine (CBZ)-nicotinamide (NCT) cocrystal formation facilitated by moisture sorption by Poly Vinyl Pyrollidone K12 (PVP K12). As PVP K12 decreased the ratio of cocrystal to drug solubility, increase in PVP K12 concentration increased the stability of carbamazepine (CBZ)-nicotinamide (NCT) cocrystal [160]. Figure 11 represents the optical microscopic images showing carbamazepine-nicotinamide cocrystal formation by moisture sorption, dissolution, cocrystal nucleation and growth in presence of PVP K12 as a deliquescent polymer at 75% RH and 25 °C.

### 5.2. Continuous Production of Cocrystals

Continuous production of cocrystals has gained much attention from pharmaceutical sectors [179,180,181,182]. In addition to enabling large-scale production of cocrystals, continuous production methods also offer significant advantages over batch processes [182] as mentioned below:

- Batchwise variation in cocrystals quality can be rectified by means of continuous production by adopting a Quality-based Design (QbD) approach
- Continuous production of cocrystals is less labor intensive when compared to batch mode
- As compared to batch process, the maintenance of uniform particle distribution throughout the entire production, monitoring of quality of cocrystals by Process Analytical Technology (PAT) and maintaining consistency in the quality of products obtained is easier for continuous production

Examples of different continuous cocrystal production methods are Twin Screw Extrusion [183], Hot-Melt Extrusion [65,184], Co-Extrusion, Solvent-Free Continuous Cocrystallization [185], Spray Drying [186], Spray Flash evaporation process [187], Solid-State Shear Milling Technology [188], melt crystallization [48] and melt-assisted grinding [179]. Table 9 presents a summary of a few literature reports available on continuous cocrystallization production techniques. Solid-State Shear Milling Technology [188], melt crystallization [48] and supercritical fluid-based methods [189,190] are discussed in detail in the following Section 5.2 (a–c).

Though continuous cocrystallization production methods can be employed to give high yield of cocrystals, these methods have certain limitations in terms of reproducible cycling efficiency and accurate process control. Maintaining stability and quality of products when transferred from one unit operation to another (Quality assurance) to make these products suitable for further use can be a challenge.

**(a) Solid-State Shear Milling (S3M) Technology**

**Figure 12:**
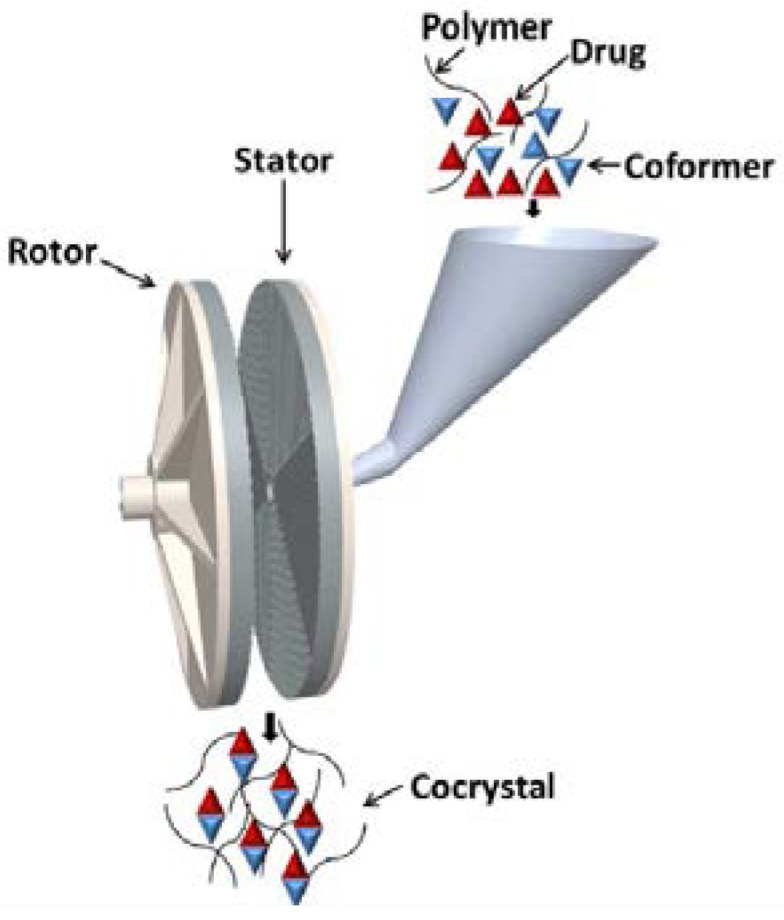
Schematic representation of ‘Solid-State Shear Milling’ technology for cocrystals production as proposed by Korde et al. [188] [Reprinted from [188] with permission. Copyright 2018 American Chemical Society].

Recently, Korde et al. [188] employed ‘Solid-State Shear Milling’ technology to produce carbamazepine-salicylic acid cocrystals with 5–25 wt. % of poly(ethylene oxide) (PEO). This ‘Solid-State Shear Milling’ technology was reported to be a scalable and polymer-assisted technology for continuous production of cocrystals [188]. Figure 12 presents the schematic of ‘Solid-State Shear Milling (S3M)’ technology employed by Korde et al. [188]. In this technique, well-mixed API and coformer are blended along with a polymer and then subjected to fine grinding by S3M. The S3M consists of two inlaid pans, a rotor and a stator which are made up of wear-resistant materials. The applied shear force for milling along with the polymeric aid facilitates efficient milling due to generation of high stress fields for grinding [188].

**(b) Melt Crystallization**

Melt crystallization has been adopted to produce cocrystals by several researchers [144,191,192]. This is an indirect way in which the cocrystal phase is formed from the melting of eutectic phase. Melting of API and coformer beyond the eutectic temperature creates a eutectic melt and the cocrystal phase growth appears from nucleation in the eutectic melt. For the drug-coformer pairs which form cocrystals from the eutectic melt, melt crystallization can yield cocrystal without any phase impurity [191,192]. Melt crystallization is a solvent-free, scalable and continuous method for cocrystal production [48].

Douroumis and coworkers [179] have contributed an extensive review on various solvent-free continuous cocrystal manufacturing methods [179]. Recently, Crawford [193] has contributed a review on the applications of Twin Screw Extrusion in continuous production of organic compounds [193]. Also, Shastri and coworkers [181] have recently provided a detailed review on the various challenges associated with continuous cocrystal production methods and the prospects in this technology [181].

**(c) Supercritical CO_2_-Based Methods**

Utilization of supercritical CO_2_ as solvent or an anti-solvent instead of using liquid solvents serves as an excellent mean for large-scale production of cocrystals [194]. Pando et al. [162] had presented an extensive review on preparation of pharmaceutical cocrystals using supercritical CO_2_ [162]. Efforts have been made by many researchers to prepare cocrystals by Gas Anti-solvent Crystallization (GAS) [189,190,194,195,196,197,198] and to compare its morphology, crystalline nature and dissolution rates with cocrystals prepared via Liquid Anti-Solvent (LAS) precipitation or traditional solution-based methods [194,195,196]. In addition to GAS, Supercritical fluid Enhanced Atomization (SEA) [190], Atomization and Anti-solvent Crystallization (AAS) [191] and Supercritical Anti-solvent crystallization (SAS) [189] are a few other techniques where supercritical CO_2_ has been utilized as an anti-solvent for cocrystals production. Rapid Expansion of Supercritical Solutions (RESS) is an interesting technique where both micronization of particles and cocrystallization can be achieved simultaneously using supercritical CO_2_ as a solvent [199]. RESS involves no toxic solvents and therefore serves as an ecofriendly technique for a large-scale production of cocrystals. While supercritical CO_2_ methods can be used for continuous synthesis of cocrystals, requirement of high pressure and especially designed nozzles for atomization can limit the usefulness of such techniques for large-scale production.

## 6. Characterization of Cocrystals

### 6.1. Structural Analysis

The crystalline nature of the cocrystals can be characterized by Powder X-Ray Diffraction (PXRD) and single crystal X-Ray Diffraction (SCXRD). While SCXRD provides detailed structural information including the lattice parameters, space group, miller indices, crystal system, unit cell volume, inter and intramolecular interactions, the PXRD provides information about the crystallinity of the solid phase. However, when the crystals produced by solution crystallization are not of good quality to conduct SCXRD analysis, one can possibly extract the structural data from PXRD data using indexing programs such as TREOR90 [200], ITO [201] and AUTOX [202], DASH [203], Rex.Cell [204] and Rietveld refinement programs such as TOPAS [205] and EXPO [206]. International Union of Crystallography (IUCr) serves as a valuable resource, containing information about different crystallographic software used for determining single crystal data of cocrystals from their corresponding PXRD data [207]. Several reports are available in the literature where researchers have solved the crystal structure of cocrystals from Powder X-Ray Diffraction data [208,209].

### 6.2. Thermal Analysis

**Figure 13:**
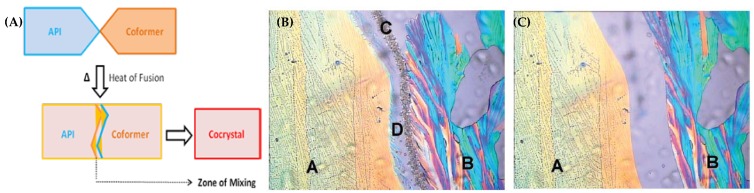
(**A**) Schematic representation of Hot-Stage Microscopy (HSM) functioning principle [210], formation of lamotrigine-caffeine (2:1) cocrystal using HSM technique. Contact area between lamotrigine and caffeine (**B**) At 190 °C [A—caffeine in solid-state; B—lamotrigine in solid state; D—Melting of eutectic and C—Formation of cocrystal phase] and (**C**) At 200 °C [A—caffeine in solid-state; B—lamotrigine in solid state; C and D observed in Figure 13B disappears due to complete melting of the cocrystal phase] [Reprinted from [211] with permission. Copyright 2012 American Chemical Society].

Hot-Stage Microscopy (HSM or Kofler method): A thermal microscopic method provides a visual understanding of phase transitions which occur while heating an API-coformer mixture to a certain temperature [210,211,212,213,214,215]. It is also called as Mixed-fusion method in which one of the components (first component) melts initially. It is then followed by the solubilization of a second component into the molten component. This complete recrystallization results in the formation of ‘Zone of mixing’ which represents cocrystal formation from eutectic melt or polymorphic transformation of cocrystals (as shown in Figure 13A). HSM was reported to be used as a cocrystal/cocrystal polymorphs screening technique in several reports [216,217].

Figure 13B,C shows an example of a cocrystal formation between lamotrigine and caffeine by using HSM [211]. Figure 13B indicates formation of cocrystal phase at 130 °C [202] followed by melting of eutectic phase at 190 °C whereas the cocrystal phase melt is observed when the temperature is increased to 200 °C (as shown in Figure 13C) [211]. The results obtained from HSM were also compared with the DSC thermograms obtained for lamotrigine-caffeine (2:1) pair. The results obtained in HSM and DSC analysis showed a good correlation between these two techniques. Thus, HSM/DSC screening method was suggested to be an efficient method for cocrystal screening when the quantity of sample available for cocrystal screening is less [211].

In addition to Hot-Stage Microscopy, Differential Scanning Calorimetry also serves as an effective thermal analysis technique for characterizing the cocrystal phases (as explained in Section 4.2).

### 6.3. Spectroscopic Analysis

There are several spectroscopic techniques which are commonly used to characterize the cocrystal phases. Fourier Transform (FT-IR) Infrared Spectroscopy (a vibrational spectroscopy) is a non-destructive analysis technique used for identifying hydrogen bonds and molecular conformations in a cocrystal phase with respect to its pure components based on the differences in the vibrational modes exhibited by the samples due to absorption of light.

Fourier Transform Near-Infra Red (FTNIR) Spectroscopy is a non-destructive vibrational spectroscopic technique which records the vibrational changes in the near-infra red region due to absorption of light by vibrating molecules. It is used to detect hydrogen bonds in a cocrystal phase based on the spectral changes seen in a spectrum of a cocrystal phase in comparison to near-infrared spectrum obtained for the parent molecules [218].

Raman spectroscopy is also a non-destructive spectroscopic technique used to detect the vibrational changes in the spectrum of a cocrystal phase which occurs due to scattering of incident light. Furthermore, through solid-state Nuclear Magnetic Resonance (ssNMR) spectroscopy, one can obtain detailed structural information of pharmaceutical solid phases such as cocrystals, eutectics and amorphous solids [219]. ssNMR spectroscopy provides information regarding molecular mobility, differences in hydrogen bonding and crystallinity of solids. The cocrystal powders which are difficult to be characterized by single crystal X-Ray diffraction analysis (due to poor crystal quality) to obtain a crystal structure can be characterized by solid-state Nuclear Magnetic Resonance (ssNMR) spectroscopy [219,220].

Proton NMR spectroscopy is a nuclear magnetic resonance spectroscopy which can be used for determining stoichiometric ratio of cocrystal phases [221].

Furthermore, Variable Temperature X-ray Diffraction (VTXRD) analysis is used to determine phase transformation events which occur at a specific temperature during heating of a cocrystal phase. Through VTXRD, in addition to identification of polymorphic phase transformations, one can also determine the crystalline nature of the solid phase at different temperatures. Reports are available in the literature were VTXRD has been used to determine phase transformation events observed for different [222,223]. Vangala et al. [222] reported that dimorphs of caffeine-glutaric acid (1:1) cocrystal are enantiotropically related with the transition temperature of 79 °C [222] using VTXRD studies.

In addition to the characterization techniques discussed above, some of the well-advanced techniques such as pair-distribution function PXRD [224], Terahertz (THz) spectroscopic imaging [225], Probe-Type Low-Frequency Raman Spectroscopy [226], combined near IR and Raman spectroscopies [227], in situ Raman spectroscopy, in situthermomicroscopy [192], calorimetric analysis [192] and PAT [75] are being used widely by many researchers nowadays in order to understand the phase transition events that possibly occur during a cocrystallization process.

### 6.4. Morphological Characterization

Polarized optical microscopy [228], Fluorescence microscopy [229] and Scanning Electron Microscopy (SEM) [24,147,217] are the various techniques which can be used to characterize the morphology of cocrystals.

### 6.5. Drug Release Testing

According to United States Food and Drug Administration (US FDA), to ensure that pure drug is released from the cocrystal in vivo, evaluation of the performance of cocrystals by in vitro dissolution study or solubility testing is highly mandatory [230,231]. Determination of cocrystal solubility, determination of powder and intrinsic dissolution rates using United States Pharmacopeia apparatus [61,65,68,72,101] and evaluating cocrystal diffusivity by dialysis and Franz-diffusion cell method [232] are some of the in vitro drug release testing methods used for cocrystal formulations. Achieving in vitro-in vivo correlation (IVIVC) of a pure drug from its cocrystal form is an important criterion that must be satisfied by cocrystals for their use in pharmaceutical applications.

## 7. Influence of Intermolecular Interactions on Cocrystallization

The type and strength of intermolecular interactions that occur between the drug and coformer molecules determine the nature and the stability of the multicomponent solids (cocrystals/salts/coamorphous solids/eutectics/solid solutions/physical mixture) formed during cocrystallization. There are several types of intermolecular interactions (hydrogen bonding, secondary bonding, pi-pi interactions, ionic interactions, halogen bonding, vanderwaal’s forces and dipolar interactions) which can possibly exist in multicomponent systems. However, most of the definitions for cocrystals are primarily based on considering hydrogen bonding as a predominant intermolecular interaction stabilizing the cocrystal lattice [233,234,235]. In 1940, Glasstone called cocrystals as ‘*lattice compounds*’ in his book, *Textbook of Physical Chemistry* and defined them as ‘substances formed between stoichiometric amounts of “two” molecular species, which owe their stability to packing in the crystal lattice, and not to ordinary valency forces’ [235]. Later, Dunitz [236] proposed that the word ‘two’ in Glasstone’s definition should be changed to ‘two or more’ [236]. However, some of the definitions given for cocrystals by many researchers were solely based on the nature of components (gas phase/ionic molecules/solid components) which constitute the crystal lattice. Later, an unambiguous definition which covers all the characteristics of cocrystal had become necessary. Attempts were made by Lara-Ochoa and Espinosa-Perez [237] to propose a universal definition for cocrystals by considering three non-ordinary valence forces (π-π interactions and vanderwaal’s forces, π-π stacking interactions and halogen bonding) stabilizing the cocrystal structure [237].

The following are the different types of intermolecular interactions reported to exist in the crystal structure of different cocrystals:

### 7.1. Hydrogen Bonding

Hydrogen bonding is a non-covalent interaction that occurs between a hydrogen atom attached to any electronegative atom such as oxygen or nitrogen and another electronegative atom. It is commonly represented as X-H---Y where X and Y are the electronegative atoms. Hydrogen-bonding interaction can be an intermolecular or intramolecular interaction. Hydrogen bonding is a specific type of dipolar interaction that serves as a key parameter in crystal engineering approach. Most of the cocrystal structures reported in Cambridge Structural Database (CSD) are stabilized by hydrogen-bonding interactions. Hydrogen bonds are individually weak bonds but collectively they are stronger enough to form supramolecular synthons in multicomponent systems and stabilize their structures [99,238,239].

**Figure 14:**
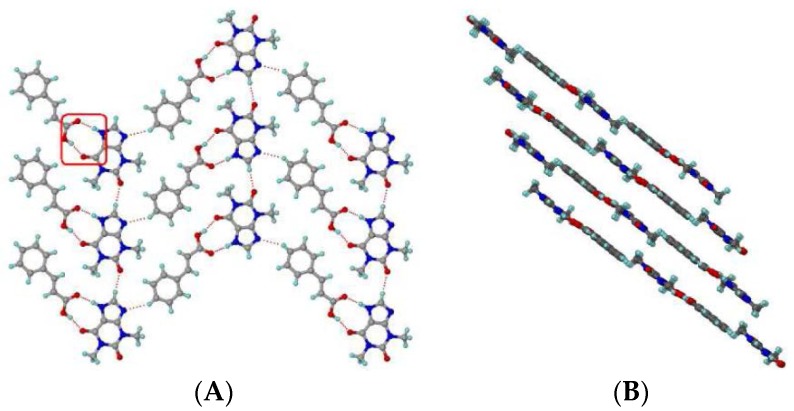
(**A**) 1D zig-zag chain arrangement brought about by 2-point acid…amide synthon through formation of O-H…O and N-H…O hydrogen bonds in theophylline-cinnamic acid (1:1) cocrystal, (**B**) Stacking of 2D layers by weak interactions completes the 3D arrangement [Reprinted from [240] with permission. Copyright 2014 American Chemical Society].

Figure 14 presents the different types of hydrogen bond synthons which were reported to be observed in theophylline-cinnamic acid (1:1) cocrystals [240]. The crystal structure obtained for theophylline-cinnamic acid (1:1) cocrystal (Figure 14) showed that cinnamic acid forms a carboxylic acid two-point synthon with carbonyl O of the pyrimidine ring and with the N–H hydrogen of the imidazole ring of theophylline to stabilize the crystal structure. This kind of stronger interaction prevents the formation of cocrystal hydrate [240].

### 7.2. Halogen Bonding

**Figure 15:**
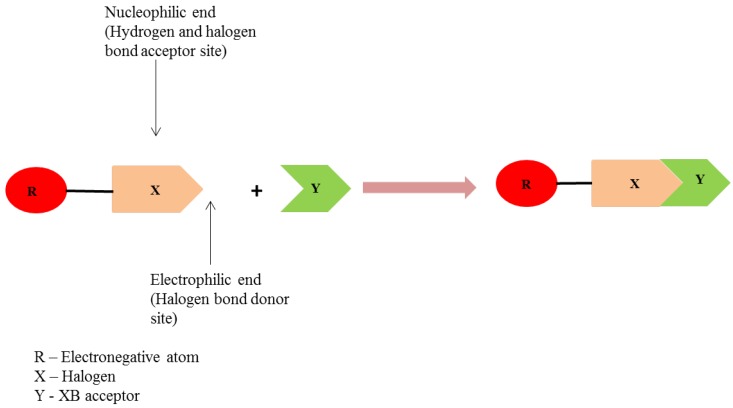
Schematic representation of halogen bonding between two molecules [241].

In recent years, halogen-bonded cocrystals have gained an extensive attention. Figure 15 presents the schematic of halogen bonding interaction between two molecules.

Halogen bonds are more directional than hydrogen bonds [242] but are analogous to hydrogen bonds [243]. Novick et al. [244] stated that halogen bonds are ‘anti-hydrogen bonds’ [245] while Alkorta et al. [245], Desiraju and Steiner [246] called halogen bonds as ‘inverse hydrogen bonds’ [245,246] to emphasize the difference between the hydrogen and halogen bonding. Since the halogen atom attached to an electronegative atom (a molecule with ‘R’ group) contains both nucleophilic and electrophilic end, it can interact with neighboring molecule to form non-covalent bonding, in turn contributing to the building of a new supramolecular structure (as shown in Figure 15).

**Figure 16:**
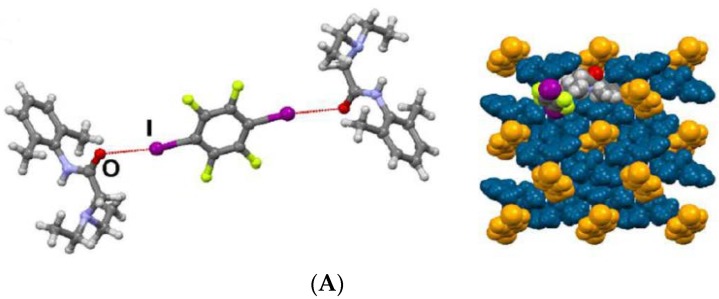

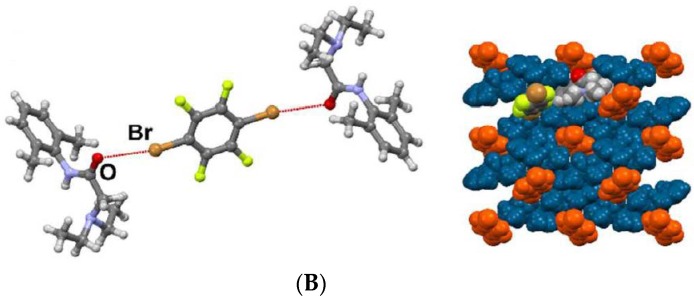
Supramolecular synthons observed in crystal structures of (**A**) lidocaine-1,4-diiodotetrafluorobenzene (2:1) cocrystal, (**B**) lidocaine-1,4-dibromotetrafluorobenzene (2:1) cocrystal [Yellow and orange represents halogen-bond donors; blue represents halogen-bond acceptors] [Reprinted from [247] with permission. Copyright 2017 American Chemical Society].

Choquesillo-Lazarte et al. [247] has investigated the nature of halogen bonding interactions observed in pharmaceutical cocrystals of pyrazinamide, lidocaine and pentoxifylline with perfluorinated halogen-bond donors namely 1,4-diiodotetrafluorobenzene (tfib) and 1,4-dibromotetrafluorobenzene [247]. The halogen bonding interaction observed in lidocaine-1,4-diiodotetrafluorobenzene (2:1) and lidocaine-1,4-dibromotetrafluorobenzene (2:1) cocrystals are shown in Figure 16. 1,4-diiodotetrafluorobenzene and 1,4-dibromotetrafluorobenzene are structurally similar and ditopic linear halogen-bond donors. The crystal structure of lidocaine-1,4-diiodotetrafluorobenzene (2:1) and lidocaine-1,4-dibromotetrafluorobenzene (2:1) cocrystals revealed that halogen-bond donors, I/Br form intermolecular halogen bonding interaction with carbonyl oxygen of lidocaine. Thus, the oxygen of carbonyl group in lidocaine acted as a halogen-bond acceptor, forming X…O type halogen bonding with I/Br of coformer molecules, which is again a type II halogen bonding interaction [248] (as shown in Figure 16).

### 7.3. Ionic Interactions

**Figure 17:**
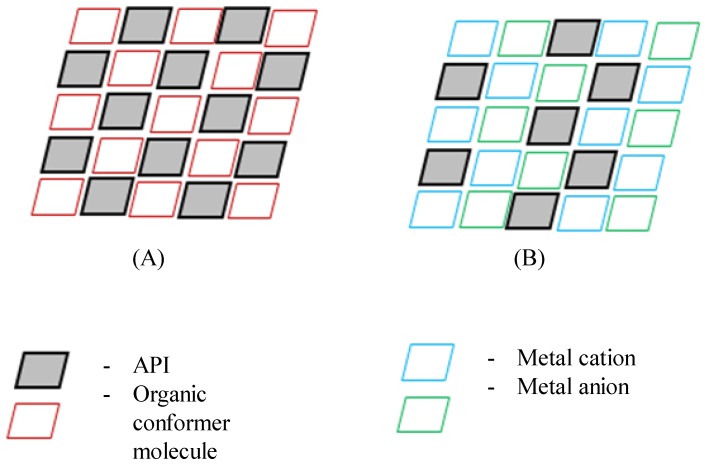
Schematic representation of (**A**) a pharmaceutical cocrystal where the API and coformer are organic molecules and (**B**) an ionic cocrystal which contains an organic molecule and a metal ion.

Ionic cocrystals are another interesting class of cocrystals. These cocrystals are formed out of organic molecules and inorganic salts [248,249]. Braga and his coworkers [250] first coined the term ‘ionic cocrystals’. Ionic interactions along with non-covalent interactions such as hydrogen bonding and dipole-bonding interactions stabilize their crystal structure [248]. However, United States Food and Drug Administration (US FDA) and EMA has declared that pharmaceutical cocrystals are crystalline materials composed of two or more components interconnected by non-ionic interactions in a crystal lattice [48,49,50]. Hence, it appears that ionic cocrystals are exception to the definition given by US FDA and EMA on pharmaceutical cocrystals. Ionic cocrystals are also referred as ‘Salt cocrystals’ when the organic molecules cocrystallize with organic salts [249]. The salt entity in ionic cocrystals can be either organic [57] or inorganic [248,250,251]. The schematic shown in Figure 17 explains a difference between an organic cocrystal and ionic cocrystal [252].

**Figure 18:**
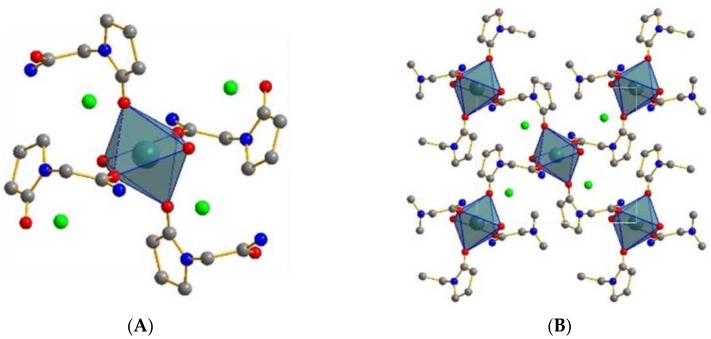
(**A**) Coordination around Ca^2+^ cation in piracetam. CaCl_2_ 2H_2_O ionic cocrystal and (**B**). 2D layer of Piracetam.CaCl_2_ 2H_2_O ionic cocrystal [Reprinted from [223] with permission. Copyright 2018 American Chemical Society].

Recently, Song et al. [223] synthesized ionic cocrystal of piracetam with calcium chloride by optimizing solvent parameters for solution crystallization through construction of Ternary Phase Diagram. The resultant ionic cocrystal had molecular formula of piracetam_2_·CaCl_2_·2H_2_O which is the piracetam dihydrate ionic cocrystal [223]. The coordination around Ca^2+^ cation in piracetam CaCl_2_ 2H_2_O ionic cocrystal and the 2D layer of Piracetam CaCl_2_ 2H_2_O ionic cocrystal have been shown in Figure 18 [223]. The piracetam CaCl_2_.2H_2_O crystallized in in a monoclinic space group, P2_1_/n. A unit cell of the ionic cocrystal consisted of two molecules of piracetam, one CaCl_2_ molecule and two H_2_O molecules [223]. Table 10 presents the summary of ionic cocrystals reported till date in the literature.

### 7.4. Π…Π Stacking Interactions

Π…Π stacking interactions are a type of attractive non-covalent interactions which occur between aromatic rings (as they contain Π bonds). The probability of occurrence of Π…Π stacking interactions increases when the number of hydrogen poor aromatic residues is more in a molecule [256]. Several reports are available in the literature where the role of hydrogen bonding in altering physiochemical properties of an API has been reported [15,95]. However, very limited reports are available in the literature where Π…Π stacking interactions were utilized for modulating the physiochemical properties of pharmaceutical cocrystals [17,257,258].

**Figure 19:**
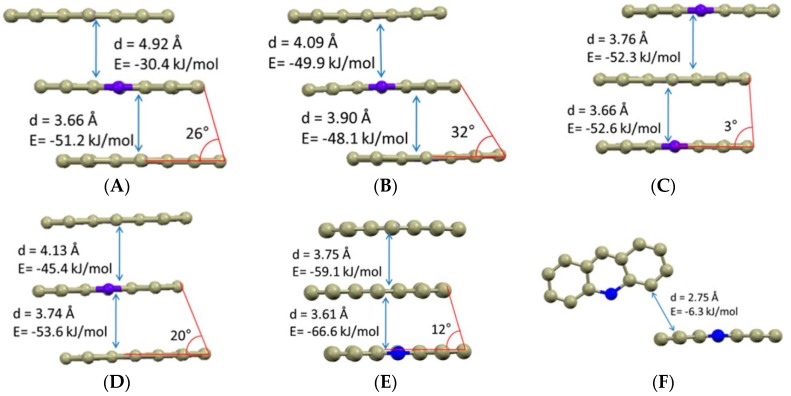
Pictorial representation of π-stacking interaction observed in (**A**) Acridine-2,3 DHBA (1:1) cocrystal, (**B**) acridine-2,4 DHBA (1:1) cocrystal, (**C**) acridine-2,5 DHBA (1:1) cocrystal, (**D**) acridine-2,6 DHBA (1:1) cocrystal, (**E**) acridine-3,5 DHBA (3:1) cocrystal and (**F**) C-H…π interaction observed in acridine-3,5 DHBA (3:1) cocrystal [Reprinted from [17] with permission. Copyright 2018 American Chemical Society].

Recently, Bora et al. [17] reported that Π…Π interactions (with T-shaped motif structures) and C−H⋯π interactions can modulate the physiochemical properties of cocrystals such as pH-dependent solubility, crystal packing and permeability while exploring cocrystallization of acridine with isomeric hydroxybenzoic acids. Since acridine actively participates in intermolecular hydrogen bonding and Π-stacking interactions (namely face-to-face π-stacking interactions and edge to face T-shaped C-H…Π interactions), it was chosen for their study [17]. It was concluded that packing of the cocrystal lattice stabilized by π-stacking interactions and C-H…Π interactions affects the solubility and membrane permeability of cocrystals. Figure 19 provides a pictorial representation of π-stacking and C-H…Π interactions observed in these cocrystal structures.

**Figure 20:**
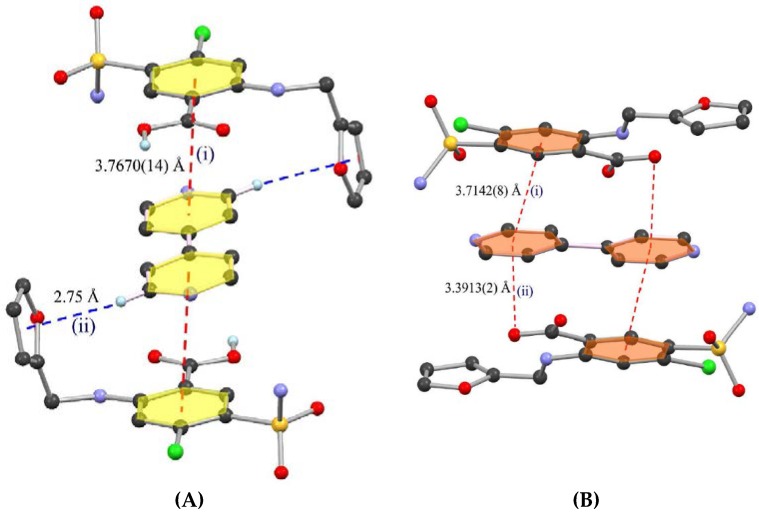
Sandwich motifs in colored furosemide-4,4′-bipyridine (2:1) cocrystal polymorphs (**A**) form I and (**B**) form IIformed viaΠ…Π interactions and C-H…Π interactions [Reprinted from [257] with permission. Copyright 2015 American Chemical Society].

Sangtani et al. [257] observed concomitant color polymorphism in Furosemide-4,4′-bipyridine (2:1) cocrystals (Forms I and II). Form I cocrystal appeared as pale yellow needles whereas form II cocrystal appeared as orange blocks [257]. Form I cocrystal was observed to crystallize at faster rate and with higher yield than the form II cocrystal. The authors proposed that the color polymorphism in furosemide-4,4′-bipyridine (2:1) cocrystals can be attributed to the difference in Π…Π^*^ separation (as shown in Figure 20) between the benzene ring of furosemide and pyridine ring of 4,4′-bipyridine in the sandwich motifs of form I and form II cocrystals [257]. This was further supported by Dynamic Functional Theory (DFT) calculations. From DFT calculations, it was evident that the Highest Occupied Molecular Orbital (HOMO)—Lowest Unoccupied Molecular Orbital (LUMO) gap for form I pale yellow needles was more when compared with form II orange blocks [257].

Thus, Π…Π interactions can significantly contribute to modification of physiochemical properties of an API molecule. Further research is needed to explore the role of Π…Π interactions in fine-tuning the solid-state properties (especially the dissolution rate, solubility, physical and chemical stability) of poorly water-soluble drugs.

### 7.5. Vanderwaal’s Interactions

Vanderwaal’s interactions are yet other type of intermolecular interactions which contribute to the packing of the cocrystal lattice [259]. It is the weakest of all intermolecular interactions. However, collectively these interactions are strong. These interactions were also observed to influence the formation of coamorphous solid dispersions to a certain extent [260].

## 8. Ternary and Quaternary Cocrystals

Ternary cocrystals and quaternary cocrystals have recently gained attention from several researchers mainly for poorly water-soluble drugs to enhance dissolution rates and aqueous solubility [261,262]. Ternary (three-component) and quaternary (four-component) cocrystals are supramolecular structures stabilized by robust synthons which build the entire crystal structure. Synthesizing ternary cocrystals is not easy when compared with the synthesis of binary cocrystals as maintenance of congruent solubility of drug and the two coformers is difficult and requires a lot of screening experiments. There is a need to understand the basic principles involved in formation of ternary cocrystals and intermolecular interactions involved in stabilizing their crystal structures [263,264,265]. Several approaches have been used by researchers to design ternary cocrystals based on crystal engineering and synthon engineering principles [265,266,267,268]. Some of the approaches have been explained below:

### 8.1. Approaches for Designing Ternary/Quaternary Cocrystals

**A. Hierarchical Fashion of Hydrogen Bond Formation**

Aakeroy et al. [266] developed an approach to design ternary cocrystals based on the two principle rules:

- Hydrogen bond formation take place in a hierarchical manner (best donor forms hydrogen with the best acceptor, the second-best donor with the second-best acceptor) [103]
- A small number of specific intermolecular interactions such as hydrogen-bonding interactions can contribute to larger stabilization energy of the molecular crystals [269]

Based on these rules, Aakeroy et al. [266] synthesized 3,5-dinitrobenzoic acid-isonicotinamide-3-methylbenzoic acid (1:1:1), 3,5-dinitrobenzoic acid-isonicotinamide-4-(dimethylamino)-benzoic acid (1:1:1) and 3,5-dinitrobenzoic acid-isonicotinamide-4-hydroxy-3-methoxycinnamic acid (1:1:1) ternary cocrystals [266].

**B. Long-range Synthon Aufbau Module (LSAM)**

LSAM has its origin from the ‘Aufbau principle’ proposed by Kitaigorodski in the year 1961 [270]. According to LSAM, the molecules which are closely-packed in one-dimensional chains, arrange themselves to form two-dimensional sheets. These two-dimensional sheets assemble and pack closely to form three-dimensional supramolecular structural crystal units. Reports are available in the literature where researchers have reported formation of binary (two-component) cocrystals [265], ternary (three-component) cocrystals [265,267,271,272] and quaternary (four-component) cocrystals [267] based on LSAM.

**Figure 21:**
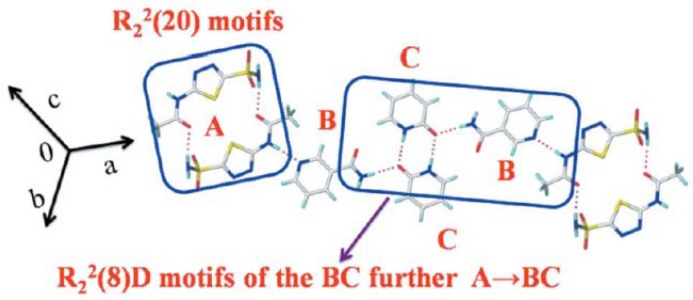
Synthons and R_2_^2^(20) motifs and R_2_^2^(8)D motifs observed in acetazolomide-nicotinamide-hydroxypyridine (1:1:1) ternary cocrystal [A—Acetazolomide; B—Nicotinamide; C—Hydroxypyridine] [Reprinted from [267] with permission of International Union of Crystallography].

**Figure 22:**
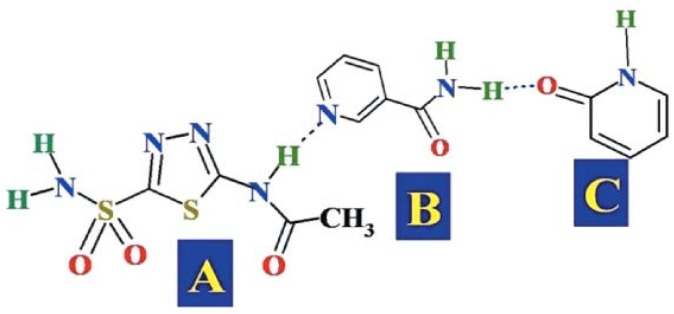
Long-range Synthon Assembly Module (LSAM) observed in acetazolomide-nicotinamide-hydroxypyridine (1:1:1) ternary cocrystal [Reprinted from [267] with permission of International Union of Crystallography].

Binary cocrystal of acetazolomide with hydroxypyridine (1:2) was synthesized based on LSAM [267]. Bolla and Nangia [267] had synthesized ternary cocrystals of acetazolomide by replacing one molecule of hydroxypyridine with nicotinamide (in acetazolomide-hydroxypyridine (1:2) binary cocrystal) and attempted to understand the LSAM [267]. Cocrystallization of acetazolomide with nicotinamide and hydroxypyridine resulted in a ternary cocrystal at a stoichiometric ratio of 1:1:1 (as shown in Figure 21). Hydrogen bonding between sulfonamide N-H and nicotinamide –C=O resulted in R_2_^2^(20) motifs whereas R_2_^2^(8)D motifs were observed in hydroxypyridine dimers (as shown in Figure 21). Thus, –NH functional group in the acetazolomide bonds with pyridine N atom of nicotinamide and –NH of nicotinamide is bonded to hydroxypyridine dimers to result into a cocrystal adopting Long-range Synthon Aufbau principle (Figure 22) [267]. Figure 22 represents the LSAM observed in acetazolomide-nicotinamide-hydroxypyridine (1:1:1) ternary cocrystal.

**C. Drug-Bridge-Drug Ternary Cocrystallization Strategy**

**Figure 23:**
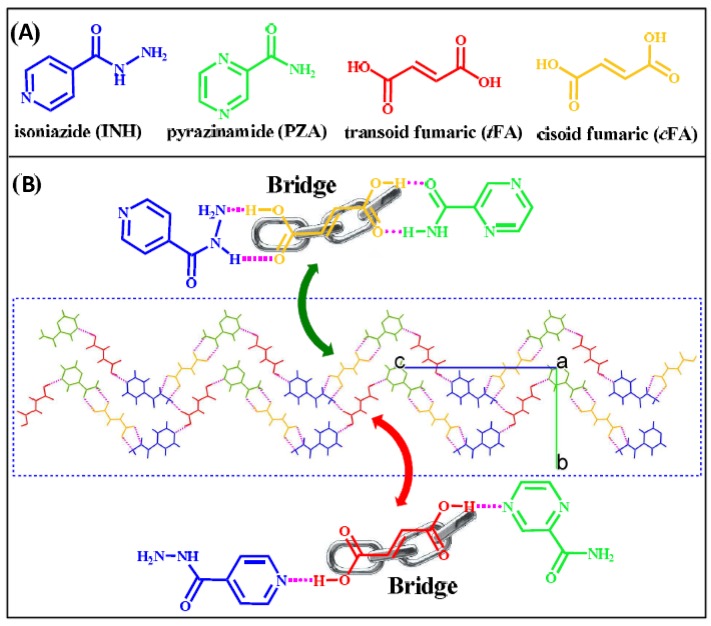
(**A**) Chemical structures of isoniazid, pyrazinamide, *trans* and *cis* fumaric acid, (**B**) Schematic representation of fumaric acid acting as a bridge in interconnecting isoniazid and pyrazinamide molecules [Reprinted from [261] with permission. Copyright 2018 American Chemical Society].

Recently, Liu et al. [261] developed a ‘Drug-Bridge-Drug Ternary Cocrystallization strategy’ for cocrystallizing two anti-tuberculosis drugs, isoniazid and pyrazinamide using *trans-*fumaric acid as a bridge. Pyrazinamide has aqueous solubility of 15,000 mg/L [273] whereas isoniazid has aqueous solubility of 140,000 mg/L at 25 °C [274]. Figure 23 presents (a) the chemical structures of the API molecules, isoniazid and pyrazinamide, *trans* and *cis* fumaric acid and (b) the schematic how fumaric acid act as a bridge in interconnecting isoniazid and pyrazinamide molecules.

Fumaric acid was chosen as a bridge to link isoniazid and pyrazinamide as the carboxylic acid functional groups in fumaric acid can interact effectively with *N*-heterocycles of isoniazid and pyrazinamide to form stronger heterosynthon [261]. Moreover, the amide functional group of pyrazinamide and hydrazide functional group in isoniazid has been reported to form hydrogen-bonding motifs with dicarboxylic acids and tricarboxylic acids in several studies [275,276]. The selection of suitable dicarboxylic acid plays an important role in determining the success of this ‘drug-bridge-drug ternary cocrystallization strategy’.

**D. Ditopic Hydrogen Bond Donors and Acceptors Combination**

**Figure 24:**
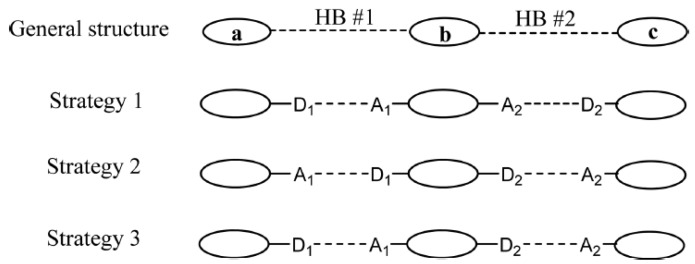
Schematic representation of possible structures of hydrogen-bonding interactions in ternary cocrystals [HB # 1 and HB # 2 -Hydrogen bonds 1 and 2; A_1_,A_2_-Hydrogen bond acceptors and D_1_,D_2_-Hydrogen bond donors] [Reprinted from [264] with permission. Copyright 2016 American Chemical Society].

Etter’s rule of hydrogen bonding [103] suggests that design of a cocrystal mainly depends on hydrogen bond acceptors and hydrogen bond donors which participate in cocrystallization (discussed in Section 3.2). In general, ternary cocrystals are stabilized by strong intermolecular interactions that exist between three components. Later, Aakeroy et al. [266] proposed that these intermolecular hydrogen-bonding interactions in ternary cocrystals can occur as per any one of the patterns illustrated in Figure 24.

**Figure 25:**
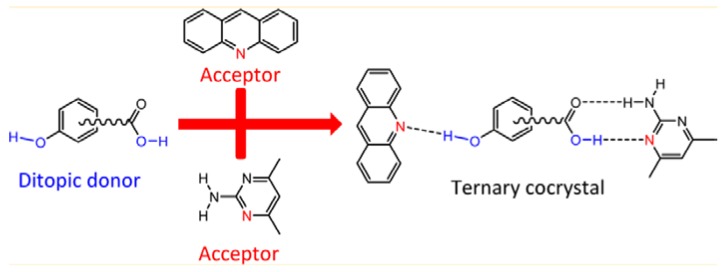
Schematic representation of ditopic hydrogen bond donors and acceptors combination strategy proposed by Adsmond et al. (2016) in formation of acridine-3-hydroxybenzoic acid-2-amino-4,6-dimethylpyridine (1:1:1) cocrystal [Reprinted from [264] with permission. Copyright 2016 American Chemical Society].

Adsmond et al. [264] adopted strategy 2 (shown in Figure 25) to design ternary cocrystals [264] of acridine, 3-hydroxybenzoic acid and 2-amino-4,6-dimethylpyridine using carboxyphenol (3-hydroxybenzoic acid) as a ditopic hydrogen donors molecule. According to the proposed strategy, a molecule with ditopic hydrogenbond donors (such as carboxy phenols) can be combined with two different molecules containing hydrogenbond acceptors in them to generate a new three-component crystal [264]. The schematic representation of the strategy used by Adsmond et al. [264] in formation of acridine-3-hydroxybenzoic acid-2-amino-4,6-dimethylpyridine (1:1:1) cocrystal is shown in Figure 25.

Also, Bhogala and Nangia [277] synthesized ternary and quaternary cocrystals of 1, 3cis,5cis-cyclohexanetricarboxylic acids with 4,4′-bipyridine bases connected by methylene and alkene chains [277]. It was concluded from the study that utilization of triacid and differentiated bipyridine bases is responsible for the self-assembly of the different components through O-H…N hydrogen bonds in the ternary and quaternary systems. Tilborg et al. [278] reported a three-component cocrystal of 1:1:1 l-proline-d-proline-fumaric acid with a racemic compound, *DL*-proline and fumaric acid [278]. Interestingly, a 1:1 eutectic mixture of Pyrazinamide-Isoniazid [18] yielded two different ternary cocrystals with succinic acid and fumaric acid as coformers (each with a stoichiometric ratio of 1:1:1) [23]. Also, Cheung et al. [279] reported that solid-state ^13^C NMR can serve as a direct method to determine the structure of ternary cocrystal of benzoquinone (BQ), *racemic* bis-β-Naphthol (BN) and anthracene (AN) in a stoichiometric ratio of 1:1:0.5, prepared by solid-state grinding [279]. Recently, ternary cocrystals of Bumetanide with isonicotinamide-2-picolinic acid, isonicotinamide-vanillic acid, isonicotinamide- *para* aminosalicylic acid and 2-hydroxypyridone-2-picolinic acid pairs were reported by Allu et al. [280]. Aitipamula et al. [262] prepared ternary cocrystals of isoniazid with enhanced dissolution rates as compared to the commercially available isoniazid [262].

### 8.2. Advantages of Ternary/Quaternary Cocrystals

Ternary/quaternary cocrystals are highly useful in enhancing the efficacy of drugs when it is not achieved by formulating binary cocrystals. Furthermore, when it is not possible to cocrystallize two different APIs (to enhance their efficacy) due to their shape incompatibility; another molecule(s) can be introduced between the two drugs which can serve as a linker, propagate the growth unit and enable cocrystallization, and form a supramolecular structure [261]. Like binary cocrystals, three- or four-component cocrystals enhance dissolution rate [261,262], bioavailability and physical stability of poorly water-soluble drugs

### 8.3. Disadvantages of Ternary/Quaternary Cocrystals

Though ternary/quaternary cocrystals possess advantages such as enhanced bioavailability, solubility, dissolution rates and physical stability, the synthesis of ternary cocrystals is difficult as the intermolecular interactions in the ternary cocrystals should be very specific and balanced. Therefore, the synthesis of ternary cocrystals involves a lot of efforts in screening and synthesis and poses difficulty in terms of understanding of the chemistry.

## 9. Polymorphism in Cocrystals

Polymorphism can be defined as the ability of a drug to exists in more than one crystalline phases with variation in arrangements or variation in the conformation of drug molecules in a crystal lattice [102,281,282]. Drugs existing in more than one crystal form can have different physicochemical properties such as dissolution rate, solubility, morphology, mechanical properties and physicochemical stability [283,284]. Polymorphism in cocrystals can influence aqueous solubility, dissolution property and bioavailability of a drug [35].

Cocrystals exhibit different types of polymorphic behaviors such as synthon polymorphism [285,286], concomitant polymorphism [257,287,288], conformational polymorphism, packing polymorphism and pseudopolymorphism [285]. Each of these types has been discussed in brief in sections below:

### 9.1. Synthon Polymorphism

**Figure 26:**
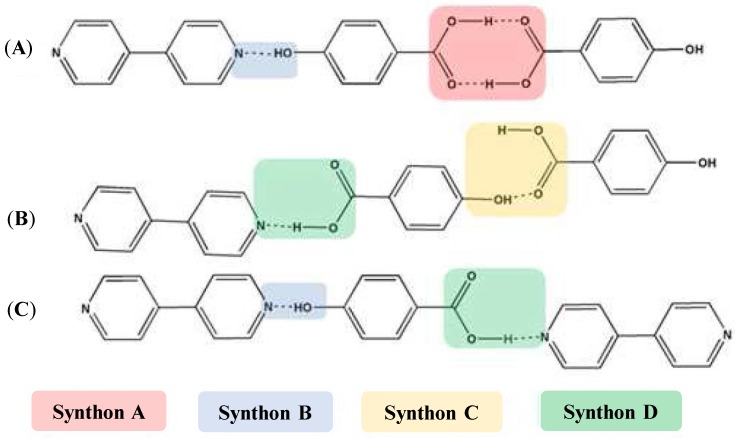
Synthon polymorphs of (**A**) form I, (**B**) form II and (**C**) form III 4-hydroxybenzoic acid:4,4′-bipyridine (2:1) cocrystal (pink—synthon A; blue—synthon B; orange—synthon C and green—synthon D) [Reprinted from [286] with permission. Copyright 2013 Royal Society of Chemistry].

Cocrystal polymorphs exhibiting difference in their synthons are termed as Synthon polymorphs [102,215,289,290,291,292,293,294,295,296,297,298,299]. Synthon polymorphs occur when a molecule has several possibilities of forming hydrogen bonds with its neighboring molecule. Figure 26 presents various synthon polymorphs (forms I, II and III) observed in 4-hydroxybenzoic acid—4,4′-bipyridine cocrystal [286]. Form I 4-hydroxybenzoic acid—4,4′-bipyridine cocrystal is stabilized by acid-acid homosynthon (represented as synthon A) and phenol-pyridine bond (represented as synthon B) and Form II 4-hydroxybenzoic acid—4,4′-bipyridine cocrystal is stabilized by phenol-acid (synthon C) and acid-pyridine bond (synthon D). Phenol-pyridine bond (synthon C) and acid-pyridine bond (synthon D) on the other hand stabilizes the cocrystal lattice of Form III 4-hydroxybenzoic acid—4,4′-bipyridine cocrystal [286].

### 9.2. Concomitant Polymorphism

Concomitant polymorphs are polymorphs which crystallize simultaneously in identical crystallization conditions in the same batch. Reports are available in the literature where researchers have reported formation of concomitant cocrystal polymorphs [285,287,288,300,301,302]. Color polymorphism is another interesting variation of concomitant polymorphism in which cocrystal forms of more than one color appear during crystallization. A few findings (See Section 7.4) on colored polymorphism of cocrystal have been reported by Gonnade and coworkers [257,258].

### 9.3. Conformational Polymorphism

**Figure 27:**
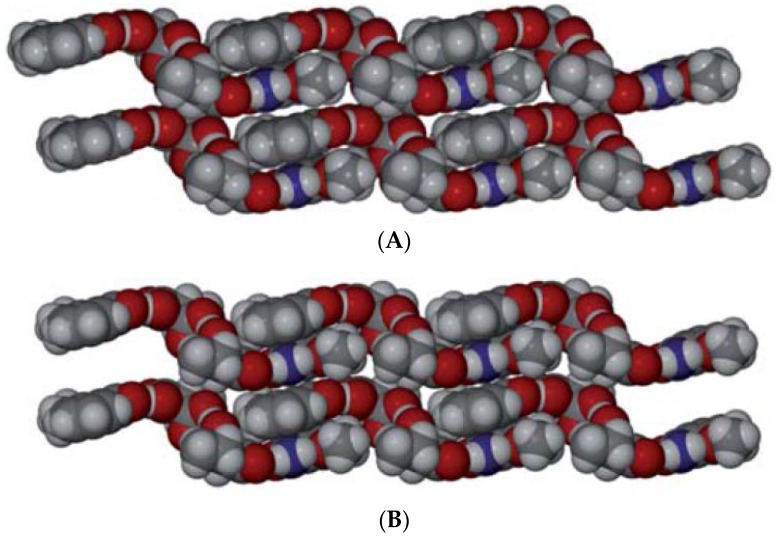
Packing of ethenzamide-ethyl malonic acid (1:1) cocrystal (**A**) form 1 and (**B**) form 2 [Reprinted from [216] with permission. Copyright 2010 Royal Society of Chemistry].

**Figure 28:**
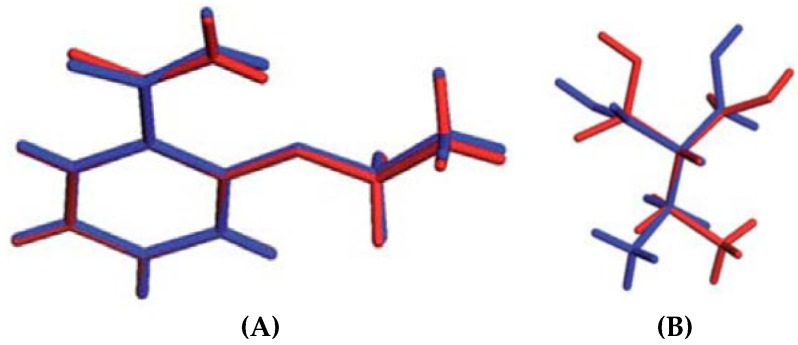
Overlay of conformers of ethenzamide (in left) and ethylmalonic acid (in right) in (**A**) form 1 (in red) and (**B**) form 2 (in blue) cocrystal ([Reprinted from [216] with permission. Copyright 2010 Royal Society of Chemistry].

When a molecule exists in different possible conformations which are formed as a result of a few rotations about a single bond present in it, then they are called as conformational polymorphs. Molecules which are conformationally flexible possess good probability to exhibit conformational polymorphism because the amounts of energy needed for rotation about single bonds are mostly equivalent to the lattice energy differences between the polymorphs [102]. Ethenzamide-ethyl malonic acid (1:1) cocrystal [294], nicotinamide-pimelic acid (1:1) cocrystal [214], caffeine-glutaric acid (1:1) cocrystal [303], trimesic acid-1,2-bis(4-pyridylethane) (2:3) cocrystal [301] and celecoxib-δ-valerolactam (1:1) cocrystal [304] were reported to exhibit conformational polymorphic behavior in the literature. Figure 27 presents the packing the of ethenzamide-ethyl malonic acid (1:1) cocrystal polymorphs, (a) form 1 and (b) form 2. The Overlay of conformers of ethenzamide and ethylmalonic acid in (a) form 1 and (b) form 2 cocrystal polymorphs are shown in Figure 28. The conformation of ethenzamide was observed to be identical in both the polymorphs (Figure 27) whereas the conformation of ethyl malonic acid differs in both (Figure 28) leading to formation of conformational polymorphs of Ethenzamide-ethyl malonic acid (1:1) cocrystal [216].

### 9.4. Packing Polymorphism

Packing polymorphs possess same conformation but different intermolecular interactions, therefore resulting in different packing patterns in the crystal lattice. When two cocrystal molecules have same conformation and different intermolecular interactions, then the cocrystals are said to be packed polymorphic cocrystals. Packing polymorphism is one of the rare polymorphic behaviors exhibited by cocrystals and very limited reports are available in the literature on packing polymorphs of cocrystals [302,305,306,307]. Skovsgaard and Bond [305] reported packing polymorphs (forms I and II) in benzoic acid-2-aminopyrimidine (2:1) and salicylic acid-*N*,*N*′-diacetylpiperazine (2:1) cocrystals [305]. Bis et al. [302] reported packing polymorphism in 4-cyanopyridine-4,4′-biphenol (2:1) cocrystals [302].

**Figure 29:**
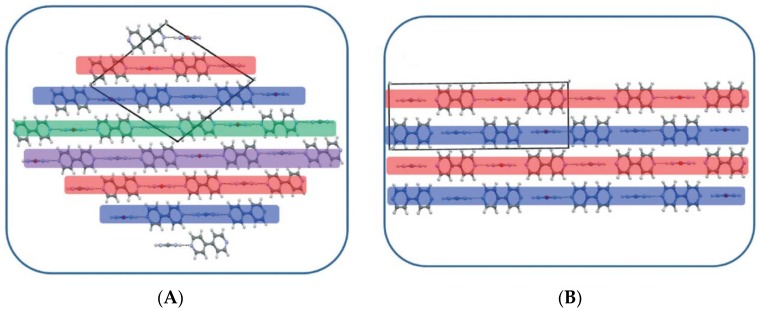
Packing of (**A**) form I urea-4,4′-bipyridine (1:1) and (**B**) form IIurea-4,4′-bipyridine (1:1) cocrystals [Reprinted from [306] with permission. Copyright 2014 Royal Society of Chemistry].

Tothadi [306] identified an interesting packing polymorphism in urea-4,4′-bipyridine (1:1) cocrystals [306]. Figure 29 presents the packing polymorphs (Form I and II) of urea-4,4′-bipyridine (1:1) cocrystals. Occurrence of urea tape in the crystal structure of urea-containing cocrystals is one of the rare observations in crystallography. Till date, out of 194 urea-containing cocrystal structures only 5 cocrystal structures were identified to possess urea tape structures [308]. Interestingly, both the polymorphic forms contained urea tapes in their crystal structures [306]. Though the urea tape arrangements are common in both the polymorphs, the 3D packing significantly differed from each other. In the Form 1 cocrystal, the consecutive planes are arranged in an ABA’B′ABA′B′ fashion whereas in the Form 2 cocrystal, the consecutive planes are arranged in an ABAB fashion.

**Figure 30:**
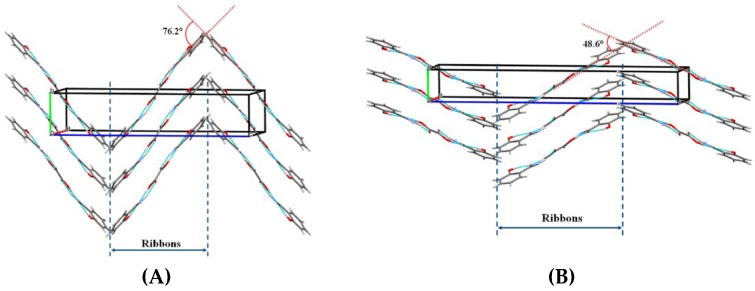
Molecular packing projections of salicylamide-oxalic acid (2:1) (**A**) form I and (**B**) form II cocrystal along crystallographic an axis [Reprinted from [307] with permission. Copyright 2017 American Chemical Society].

Recently, Surov et al. [307] addressed that weak interactions can cause packing polymorphism in pharmaceutical cocrystals while investigating packing polymorphism (form I and II) in salicylamide-oxalic acid (2:1) cocrystal [307]. The crystal structures of form I and form II cocrystal polymorphs revealed that both the polymorphs consisted of conformationally identical molecules. However, packing of the two polymorphs differed in terms of arrangement of the neighboring ribbons [307]. In form I cocrystal, the adjusted ribbons were packed in zig-zag fashion at an angle of ~76.2° while it is ~48.6° in case of form II cocrystal. Crystal structure analysis and Hirshfeld surface analysis revealed that the distribution of weak intermolecular interactions in polymorphic forms influenced the packing of the cocrystal lattice to a larger extent [307]. Figure 30 represents the molecular packing projections of salicylamide-oxalic acid (2:1) form I and form II cocrystal along an axis.

### 9.5. Pseudopolymorphism

Hydrates/solvates of an organic compound are also referred to as ‘Pseudopolymorphs’ [44]. Cocrystal pseudopolymorphs are the cocrystals which comprise water or solvent molecules as inclusions in it. 4,4′-bipyridine−4-hydroxybenzoic acid (2:1) cocrystal [285], Ethenzamide-3,5-dinitrobenzoic acid (1:1) cocrystal [294], 4-*N*,*N*′-Dimethylaminopyridine−4-Methylbenzoic Acid (1:1) cocrystal [309], Gallic acid-succinimide (at different stoichiometric ratios) cocrystal solvates [288] were reported to exist as pseudopolymorphs in the literature.

**Figure 31:**
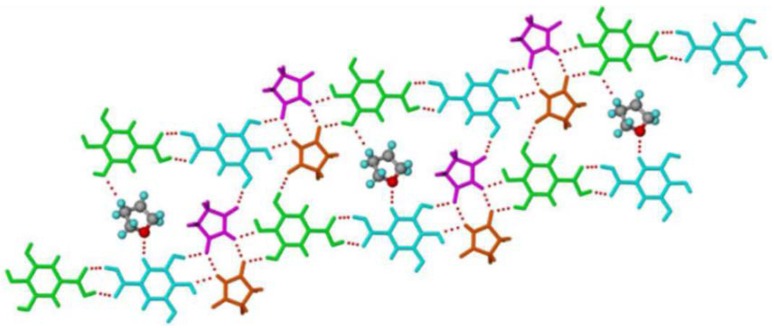
Staircase network in gallic acid-succinimide-tetrahydrofuran (2:2:1) cocrystal [Reprinted from [288] with permission. Copyright 2016 Royal Society of Chemistry].

**Figure 32:**
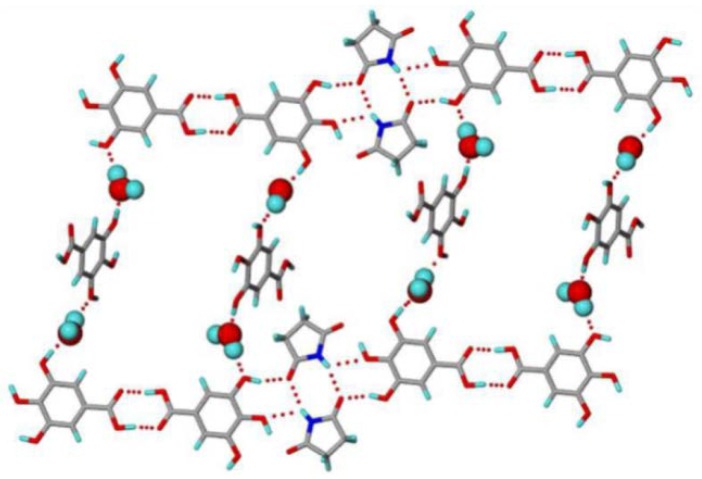
Staircase network in gallic acid-succinimide-water (1:1:1) cocrystal [Reprinted from [288] with permission. Copyright 2016 Royal Society of Chemistry].

Row and coworkers [288] reported gallic acid-succinimide cocrystal solvates/hydrates of various stoichiometric ratios formed with different types of solvents (such as 1,4-dioxane, water, tetrahydrofuran, ethyl acetate, acetone) and water inclusions as pseudopolymorphs in their study [288]. Figure 31 and Figure 32 present the staircase network of gallic acid-succinimide-tetrahydrofuran (2:2:1) cocrystal and gallic acid-succinimide-water (1:1:1) cocrystal made up of carboxylic acid and carboxamide homodimers with tetrahydrofuran and water molecules as inclusions in it. The solvent molecules occupied the voids in cocrystal lattice through C-H…O interactions (in case of tetrahydrofuran) and through O-H…O interactions (in case of water) [288].

### 9.6 Impact of Cocrystal Polymorphism on Solid-State Properties of API

Similar to the single component solids, polymorphism in multicomponent solids also plays a significant role in determining quality, efficacy and safety of an API molecule. Polymorphism has been known to influence aqueous solubility [310] and physical stability [56,230,262,310,311] of cocrystals. Paradkar and coworkers [310] synthesized dimorphic forms of carbamazepine-saccharin (1:1) cocrystal (Form I and Form II) by slow evaporative solution crystallization method and characterized their thermodynamic interrelationship [310]. During the study, it was observed from the van ‘t Hoff plot and DSC thermograms that Form I carbamazepine-saccharin (1:1) remains as a stable form whereas the Form II carbamazepine-saccharin (1:1) exists as a metastable form. Moreover, Form II polymorph showed enhanced solubility than form I polymorph with respect to different temperatures in deionized water owing to its less stability than form I [310].

## 10. Solubility and Dissolution Enhancement by Cocrystals

Bioavailability of a cocrystal is determined by its dissolution rate, aqueous solubility and permeability. Solubility is defined as a maximum amount of drug that can be dissolved in a solution and is nothing but the thermodynamic equilibrium attained by a solute between a solid and liquid phase [312]. Cocrystal dissolution refers to the amount of solute which dissolves in an aqueous medium at a specific time interval. The rate at which the solute dissolves in the aqueous medium to reach its equilibrium state is called as the dissolution rate [313]. Hence, solubility is a thermodynamic process whereas dissolution is a kinetic process. Given below is a brief account of information available on solubility and dissolution of cocrystals.

### 10.1. Cocrystal Solubilization

**Figure 33:**
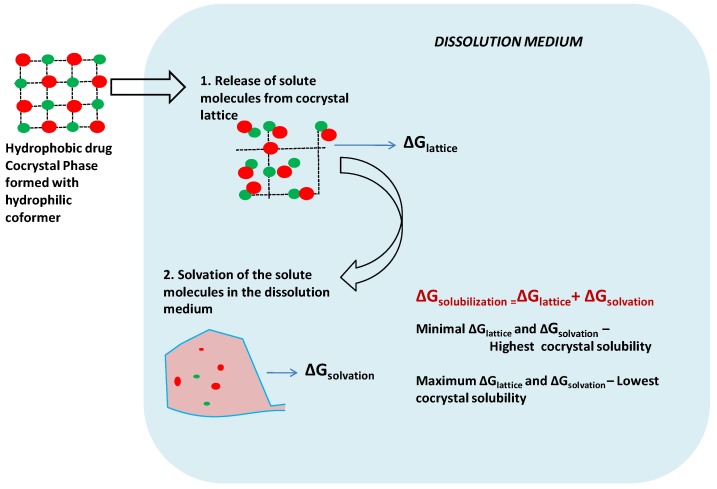
Schematic representation of the steps involved in solubilization of a hydrophobic drug in an aqueous medium.

Many researchers have attempted to understand the mechanism behind the solubility of cocrystals [9,95,314,315,316]. The insight obtained from the review of such literature reports indicates that the solubility of cocrystals depends on two important parameters namely the strength of intermolecular interactions in the crystal lattice and solvation of cocrystal components [9,10,95,314,315,316]. Maheshwari et al. [316] reported that solubilization of a cocrystal in dissolution medium involves two main steps: (1) Release of the solute molecules from the crystal lattice of the cocrystal and (2) the solvation of the released molecules [316] (as shown in Figure 33). Therefore, free energy of cocrystal solubilization depends on the free energy associated with the release of solute molecules from the cocrystal lattice and the free energy associated with the solvation barrier of the cocrystal as given in Equation (1) [316],

∆G_solution_ = ∆G_lattice_ + ∆G_solvation_ (1)

where ∆G_solution_ is the Gibb’s free energy associated with the solubilization process, ∆G_lattice_ is the Gibb’s free energy associated with the cocrystal lattice and ∆G_solvation_ is the Gibb’s free energy associated with the solvation barrier. When the free energy associated with the lattice interactions and the free energy associated with the solvation barrier becomes negligible, enhancement in cocrystal dissolution is achieved due to decrease in free energy change for solubilization [316]. The cocrystal formed with a highly water-soluble coformer, was found to possess high solubility [9,64]. Therefore, Maheshwari et al. [316] suggested that solvation is the most important barrier for the solubilization of cocrystals for hydrophobic drugs [316].

#### 10.1.1. Phase Solubility Diagram (PSD)

**Figure 34:**
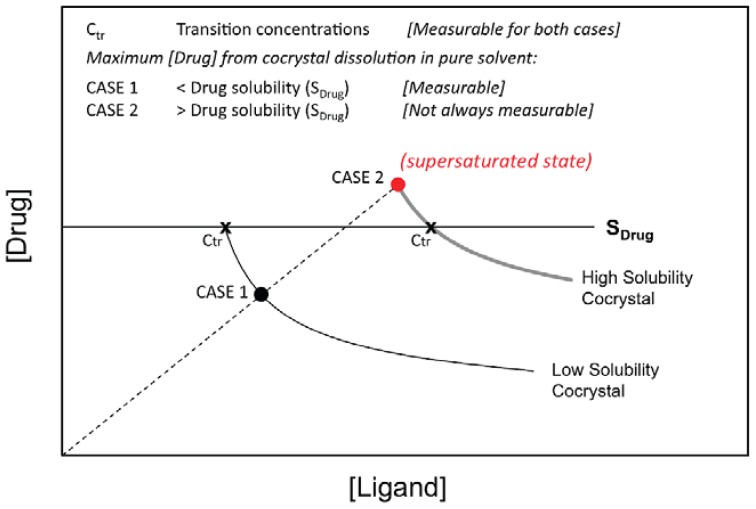
Phase Solubility Diagram explaining the solubility behavior of a low solubility cocrystal (stable cocrystal) and a high solubility cocrystal (metastable cocrystal) based on the K_sp_ value (X—transition concentrations; dashed line—stoichiometric concentrations of cocrystal components; circles—Solubility of cocrystal in pure solvent) [Reprinted from [9] with permission. Copyright 2009 American Chemical Society].

PSDs are used to identify the regions for drug and conformer stability in terms of drug and conformer concentration [9]. For a cocrystal [A_α_B_β_], drug (A) concentration can be expressed as a function of conformer (B) concentration using following equation,

[drug]^α^ = K_sp_/[coformer]^β^ (2)

where K_sp_ is the solubility product for dissolution of a cocrystal. Figure 34 is a typical PSD for two different cocrystals namely stable and metastable cocrystal [9]. In this figure, the dotted line represents stoichiometric concentrations of the drug and coformer and its intersection with the cocrystal solubility curves (denoted by filled circles) gives the maximum drug concentration corresponding to the cocrystal solubility. It can be observed from Figure 34 that the stable cocrystal exhibits lower solubility than the drug solubility in a given solvent/medium. On the other hand, a metastable cocrystal exhibits solubility higher than the drug solubility [9]. The invariant points (characterized by zero degrees of freedom and represented by x marks) shown in this figure indicate the transition points or eutectic points where solid drug, cocrystal and solution containing drug and coformer are in equilibrium [9]. Furthermore, it can be noted from Figure 34 that the stoichiometric composition of the cocrystal affects the cocrystal solubility.

#### 10.1.2. Factors Influencing Solubility of Cocrystals

During cocrystal dissolution in the aqueous medium, factors such as ionization of parent components [317], pH of the aqueous medium [317], drug-solubilizing agents [317] and coformer concentration [317] influence the solubility of cocrystals.

**(a) Ionization of Parent Components and pH of Aqueous Medium**

Cocrystal can be synthesized by cocrystallizing a neutral drug molecule with acidic or amphoteric coformer molecules or a zwitterionic drug with acidic coformer or a basic drug with acidic coformer molecule. As a result, the properties of a cocrystal designed for the same API but with different coformers also exhibit different solubility behavior mainly due to variation in ionization behavior of coformer molecules. Since ionization is highly dependent on pH, pH is another parameter which significantly affects the cocrystal solubility.

**Figure 35:**
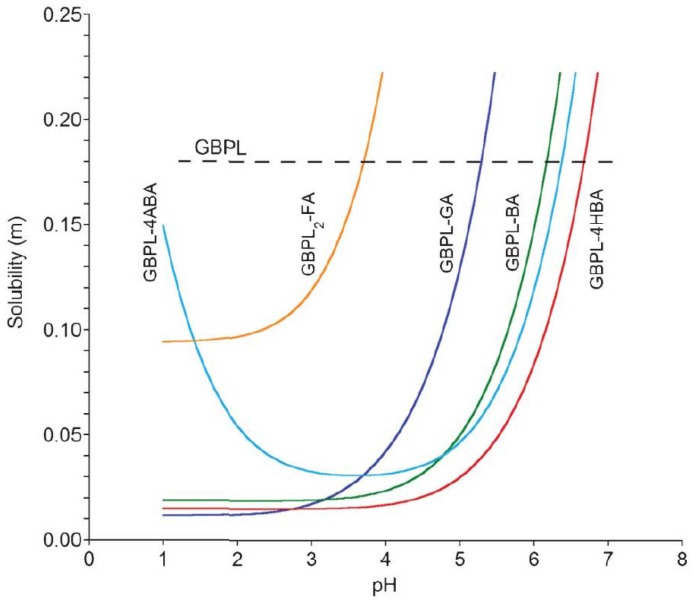
Solubility-pH profiles of Gabapentin-lactam-4-aminobenzoic acid (GBPL-4ABA), Gabapentin-lactam-fumaric acid (GBPL_2_-FA), Gabapentin-lactam-gentisic acid (GBPL-GA), Gabapentin-lactam-benzoic acid (GBPL-BA) and Gabapentin-lactam-4-hydroxybenzoic acid (GBPL-4HBA) cocrystals with respect to raw Gabapentin-lactam (GBPL) cocrystal [Reprinted from [316] with permission. Copyright 2016 Royal Society of Chemistry].

Maheshwari et al. [316] used Gabapentin-lactam (GBPL) as a model drug and synthesized its cocrystals with carboxylic acids, fumaric acid (FA), 4-hydrobenzoic acid (4HBA), genitisic acid (GA), 4-aminobenzoic acid (4ABA) and benzoic acid (BA) as coformers by reaction crystallization. The aim was to understand the influence of coformers with low aqueous solubility on the solubility of cocrystals [303]. From their study, it was identified that the so formed GBPL_2_-FA (2:1), GBPL-4HBA (1:1), GBPL-GA (1:1), GBPL-4ABA (1:1) and GBPL-BA (1:1) cocrystals showed lower aqueous solubility than raw GBPL [316] and exhibited pH-dependent solubilities. Figure 35 presents variation in solubility of gabapentin-lactam cocrystals as a function of pH. The pH value at which the solubility curve of the cocrystal and drug intersects is called as pH_max_. Figure 35 shows the pH_max_ values exhibited by five different gabapentin-lactam cocrystals [316]. Below pH_max_ value, the five GBPL cocrystals showed lower solubilitythan the drug. Therefore, this point is called as ‘the Eutectic point’ or ‘transition point’ [9,318]. The pH_max_ is also called as Gibb’s pH as it defines the thermodynamic stability of the cocrystal and above this pH the cocrystal is unstable [316].

Thakuria et al. [314] has provided a detailed review on how the ionization of components affects the pH solubility profile of different drug pairs reported in the literature. Table 11 presents generalized observations reported in the literature about the solubility behavior of cocrystals.

**(b) Effect of Solubilizing Agent**

**Figure 36:**
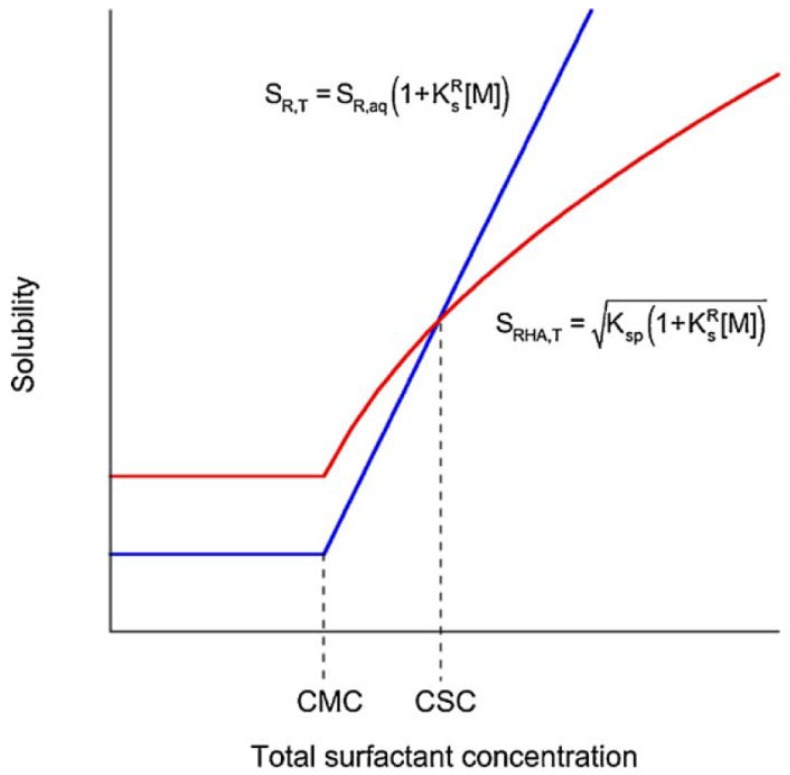
Determination of Critical Stabilization Concentration from the total surfactant concentration [Reprinted from [314] with permission from Elsevier].

Though drug-solubilizing agents such as surfactants can increase the solubility of a cocrystal in an aqueous medium [322], at a certain concentration level it also reduces the solubility of a cocrystal. The concentration of the surfactant [M] therefore determines the solubility and stability of the cocrystals in anaqueous medium. The cocrystal solubility is directly proportional to the square root of [M] whereas the drug solubility is directly proportional to [M] [314]. The concentration of surfactant at which solubility of a cocrystal and drug becomes identical is called as Critical Stabilization Concentration (CSC). The cocrystal becomes thermodynamically unstable below the CSC in the dissolution medium (See Figure 36). On the other hand, it remains in equilibrium with pure drug at CSC (this point is known as ‘the eutectic point’’) and remains thermodynamically stable above CSC. Therefore, CSC serves as an important parameter in influencing the solubility of the cocrystals.

### 10.2. Dissolution of Cocrystals

**Figure 37:**
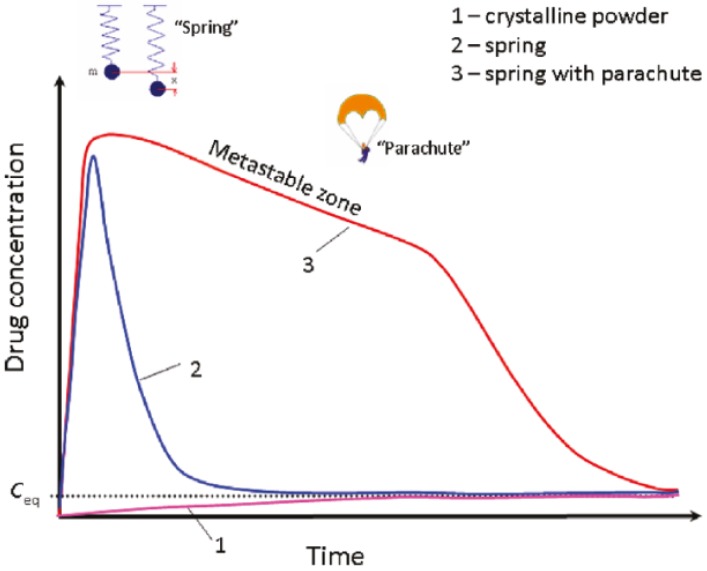
‘Spring and parachute model’ proposed to explain high apparent solubility of poorly water-soluble drugs [Reprinted from [95] with permission. Copyright 2011 American Chemical Society].

Figure 37 shows typical dissolution profiles of insoluble drugs [85] which can be characterized by “spring and parachute model” [323]. Babu and Nangia [85] stated that dissolution of cocrystals can also be explained using ‘Spring and parachute model’.

**Figure 38:**
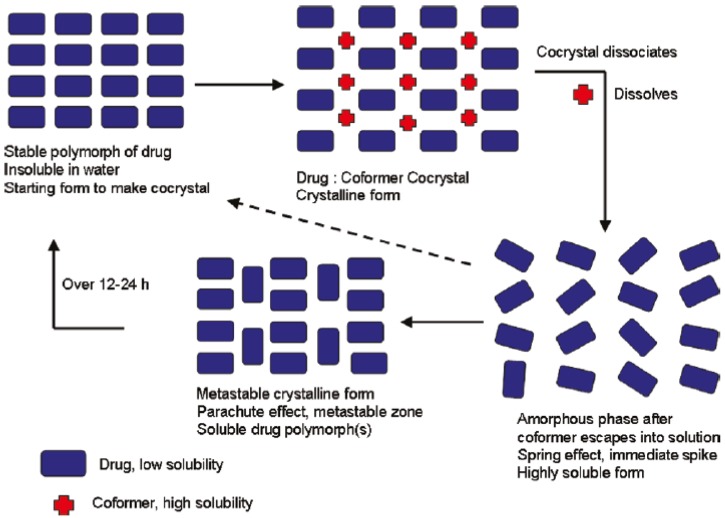
Proposed mechanism for dissolution of pharmaceutical cocrystals [Reprinted from [95] with permission. Copyright 2011 American Chemical Society].

During dissolution, cocrystals exhibit maximum peak value in the drug concentration which is attained in a shorter time (such as less than 30 min) (spring effect). This high concentration is maintained for a longer period before ultimately decreasing to the equilibrium solubility level (parachute effect) [95]. The spring and parachute effect can be explained using a mechanism proposed by Babu and Nangia [95]. As per the proposed mechanism, three main steps are involved during dissolution of cocrystals (as shown in Figure 38):

(i) Dissociation of the cocrystal into amorphous or nanocrystalline drug clusters, which is represented as ‘spring’ effect (Figure 37 and Figure 38)
(ii) Transformation of the amorphous or nanocrystalline drug clusters into a stable form through formation of a metastable phase by adopting Ostwald’s Law of Stages
(iii) Attainment of higher apparent solubility and maintenance of optimal drug concentration in the aqueous medium, which is represented as ‘parachute’ effect (Figure 37 and Figure 38).

#### 10.2.1. Factors Influencing Dissolution of Cocrystals

Dissolution of cocrystals can be influenced by several factors such as aqueous solubility of coformers (as discussed in Section 10.2.2), intermolecular interactions in cocrystals (as discussed in Section 10.2.2), crystal habit of cocrystals (as discussed below) and pH of the dissolution medium (as discussed below).

**(a) Crystal Habit**

Crystal habit of cocrystals can also influence its dissolution in dissolution medium. A drug can cocrystallize with a coformer molecule in various sizes and shapes depending on different crystallization conditions. A crystallization event can change crystal properties such as habit, polymorphism and size [324]. The term ‘crystal habit’ is used to describe the general shape of a crystal. Modification of a drug crystal’s habit during crystallization can alter its dissolution behavior due to a change in the nature of crystal faces exposed to the dissolution medium. However, studies related to the modification of cocrystal habits and understanding the effect of cocrystal habits on its dissolution properties are limited [325]. Sulfadimidine (SDM)-4-aminosalicylic acid (4-ASA) cocrystal (in 1:1 molar ratio) [polymorph I and polymorph II] were cocrystallized in four different crystal habits. These habits were obtained by solvent evaporation using ethanol (habit I) and acetone (habit II), solvent evaporation followed by grinding (habit III) and spray drying (habit IV) [See Table 12] [325]. It was observed that cocrystals prepared by milling showed highest dissolution than the cocrystal powders prepared via solvent evaporation and spray drying (as shown in Table 12). Milling produced cocrystals of very fine (smaller) particle size thereby increasing its surface area, increasing its flowability and enhancing its dissolution. On the other hand, powders prepared by solvent evaporation exhibited comparatively lesser dissolution than the former. Despite smaller particle size and higher surface area, cocrystals prepared by spray drying process showed lower dissolution which was attributed to the agglomeration of particles [325]. Therefore, Serrano and coworkers [325] suggested that crystal habits of cocrystals with poor pharmaceutical characteristics can be engineered to alter dissolution, flowability and compaction behavior [325].

**(b) pH**

Cao et al. [326] showed that the dissolution behavior of cocrystals with ionizable components is highly dependent on the interfacial pH at the dissolving solid-liquid interface [326]. When a cocrystal containing non-ionizable drug and ionizable coformer is dissolved in a dissolution medium, the dissociation reaction can modify pH at the solid-liquid interface. Authors studied the dissolution behavior of carbamazepine-saccharin and carbamazepine-salicylic acid cocrystals [326] in a dissolution medium (dissolution medium was prepared by dissolving Sodium Lauryl Sulphate (SLS) in water) with varying pH such as 1.27, 2.16, 3.02, 4.03, 5.97 and 7.66. The dissolution rate of the cocrystals was found to increase with increase in pH [326]. The mechanism of cocrystal dissolution was explained by the process of interfacial mass transport. Interfacial equilibrium model and surface saturation model were used to analyze the mass transport process. The dissolution rates of these cocrystals were found to be greatly influenced by interfacial pH and the bulk pH was not found to adequately explain the dissolution behavior [326].

#### 10.2.2. Cocrystals with Low Dissolution Rates

While cocrystallization can be used to enhance solubility of drugs, it can also be used to reduce the aqueous solubility of drugs [327]. Though enhanced aqueous solubility of poorly water-soluble drugs by formulating as cocrystals has been well-documented in the literature, the number of reports on cocrystals with lower dissolution rates than the raw drug is limited. The following are the case studies of such pharmaceutical cocrystals with lower dissolution rates reported in the literature:

**(a) Sulfacetamide Cocrystals**

Sulfacetamide is a topical antibiotic used to treat conjunctivitis. Despite its therapeutic efficacy, its pharmaceutical use is limited because of physiological constraints such as tear flow, reflex blinking and drug loss. This drawback can possibly be eradicated by frequent dosing. However, frequent dosing in turn can lead to excess drug loading in patients. Therefore, reducing the aqueous solubility of sulfacetamide can help in getting rid of excess drug loading in patients without reducing the frequency of drug administration. Therefore, Nangia and coworkers [327] adopted cocrystallization approach to bring down the aqueous solubility of sulfacetamide [327]. Sulfacetamide-caffeine (1:1) and sulfacetamide-isonicotinamide (1:1) cocrystals exhibited 0.68 and 0.64 times lower dissolution rates than raw sulfacetamide whereas sulfacetamide-theophylline (1:1) cocrystal exhibited dissolution rate equivalent to that of raw sulfacetamide [327]. It was proposed that modification of intermolecular interactions (hydrogen bonding) existing in the parent drug molecule by means of cocrystallization resulted in better crystal packing, stable crystal lattice and increase in density of the cocrystals. These modifications in the crystal lattice were responsible for lower dissolution of the synthesized sulfacetamide cocrystals, which in turn can increase the residence time of sulfacetamide drug at the site of action [327].

**(b) Fluoxetine HCl-Benzoic Acid (1:1) Cocrystal**

Fluoxetine HCl is an antidepressant drug available in the market with the trade name, Prozac. During a study carried out by Childs et al. [57] to prepare cocrystals of amine hydrochlorides with organic acids by means of crystal engineering approach, it was found that Fluoxetine HCl-Benzoic acid (1:1) cocrystal exhibited lower powder and intrinsic dissolution rates than that of commercial Fluoxetine HCl [57]. However, fluoxetine HCl cocrystals prepared with other acids such as fumaric acid and succinic acid showed enhanced powder dissolution rates. Thus, it is evident that one can increase or decrease the dissolution rates of drugs by appropriate selection of coformers for cocrystallization. The reason behind the influence of the structural and thermodynamic parameters on the dissolution rates of these cocrystals remain unclear [57]. However, it is to be noticed that the dissolution rates obtained for the cocrystals were observed to be in good correlation with the aqueous solubility of coformers [57].

**(c) Curcumin-phloroglucinol (1:1) Cocrystal**

Curcumin is a natural phenolic ingredient with anticancer activity. Its therapeutic use is limited due to its poor aqueous solubility, and hence its poor bioavailability. Efforts have been made by several researchers to enhance its aqueous solubility by forming cocrystals [24,72,328]. However, cocrystallization with phloroglucinol formed curcumin-phloroglucinol (1:1) cocrystal with lower dissolution rates [73] though phloroglucinol has higher aqueous solubility.

**(d) Lamotrigine-phenobarbital (1:1) Cocrystal**

**Figure 39:**
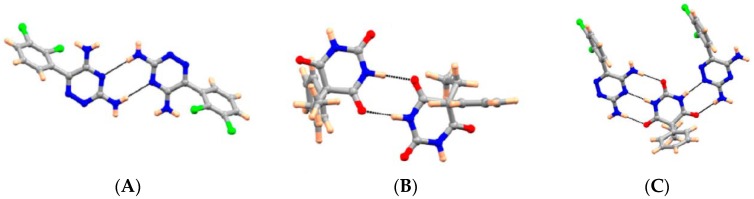
Crystal structures of (**A**) pure lamotrigine, (**B**) pure phenobarbital and (**C**) lamotrigine-phenobarbital (1:1) cocrystal [Reprinted from [329] with permission. Copyright 2017 American Chemical Society].

**Figure 40:**
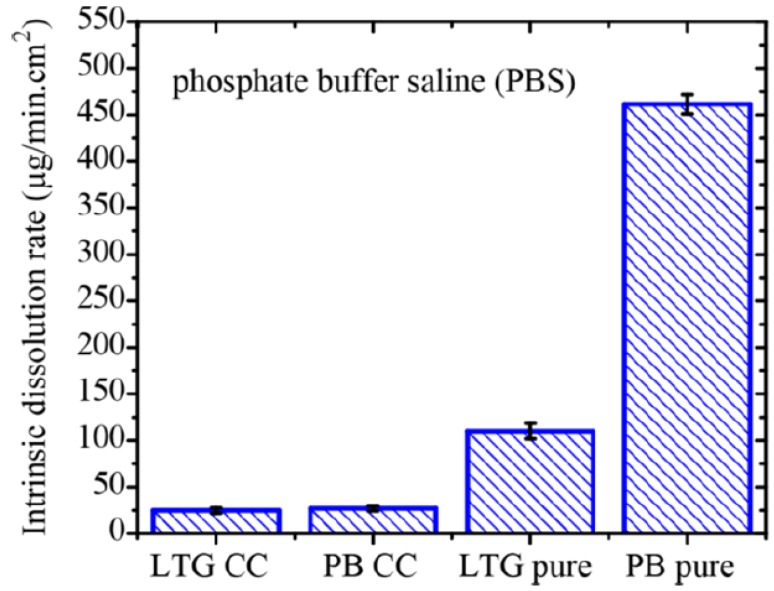
Comparison of Intrinsic Dissolution Rate (IDR) of lamotrigine from cocrystal (LTG CC), phenobarbital from cocrystal (PB CC), pure lamotrigine drug (LTG pure) and pure Phenobarbital drug (PB pure) [Reprinted from [329] with permission. Copyright 2017 American Chemical Society].

Recently, Kaur et al. [329] reported that lamotrigine—phenobarbital cocrystal (of 1:1 stoichiometric ratio) exhibited lower dissolution rate and poor aqueous solubility than raw phenobarbital and lamotrigine. Pure lamotrigine crystal is stabilized by N-H…N amide/pyridine homodimer intermolecular interactions whereas the crystal structure of pure phenobarbital is stabilized by N-H…O amine/carbonyl homodimer intermolecular interactions (as shown in Figure 39). In case of lamotrigine-phenobarbital (1:1) cocrystal, these lamotrigine and phenobarbital homodimers are held together by stronger intermolecular interactions (via formation of one N-H…N bond and two N-H…O bonds between the molecules of lamotrigine and phenobarbital) forming heterodimers (as shown in Figure 39). It was proposed by the authors that the stronger heterodimer interactions in cocrystal led to poor dissolution (as shown in Figure 40) [329].

## 11. In Vitro and In Vivo Studies

Manypharmaceutical cocrystals have been synthesized by several researchers to enhance the aqueous solubility of poorly water-soluble drugs (especially the BCS Class II and class IV drugs). However, the number of cocrystals being used as a regular drug with the approval of FDA is very low. Also, the bioavailability studies for the synthesized cocrystals have been rare. At present, Entresto (used in the treatment of chronic heart failure), Lexapro (used as an antidepressant) and Depakote (used in the treatment of seizure disorders and manic depression) are the three pharmaceutical cocrystals being approved by FDA for clinical use. Table 13 presents a summary of reports available in literature on the bioavailability assessment of pharmaceutical cocrystals. In these reports (listed in Table 13), the pharmacokinetic behavior and therapeutic effect of different solid forms have been studied. It is evident from this table that the pharmaceutical cocrystals help in fine-tuning the dissolution behavior and aqueous solubility of API molecules and in turn enhances their bioavailability. Therefore, there is a need to conduct bioavailability studies for the new solid phases (cocrystals/eutectics/coamorphous solids) prepared by cocrystallization and evaluate their efficacy in terms of their aqueous solubility under biological pH conditions, in vivo permeability and bioavailability. This would pave the way for these cocrystals to enter the next step of clinical trials for necessary approval by FDA.

## 12. Challenges and Future Perspectives

Cocrystallization of poorly water-soluble drugs is one of the novel ways to improve their aqueous solubility. A lot of research efforts are now focused on synthesizing cocrystals of poorly water-soluble drugs with appropriate coformers. The physicochemical properties of cocrystals such as melting point, aqueous solubility and hence their bioavailability depends upon the type of coformer used. Also, as mentioned earlier, the cocrystallization attempts do not always lead to a successful formation of cocrystals. At times, a eutectic or even a coamorphous solid is obtained. Therefore, choosing a correct coformer is of utmost importance. However, at present, conformers are either chosen based on empirical understanding or based on cumbersome methods requiring detailed analysis and calculations. Therefore, development of a new and fast coformer screening tool is necessary to screen coformers suitable for cocrystallization. Furthermore, efforts are also needed to develop a generalized understanding of intermolecular interactions that influence the cocrystallization outcome by employing supramolecular chemistry and crystal engineering principles. While a rationale design of a cocrystal can lead to a successful outcome at the end of cocrystallization, it is equally important to develop solvent-free cocrystal production methods such as melt crystallization. At present researchers are trying to develop continuous techniques for cocrystal synthesis. Further efforts in this direction will enable a large-scale production of cocrystals. Additionally, future research also needs to focus on stability of cocrystals. At present, very little information is available on the aspects related to the cocrystal stability such as conversion of cocrystals to drug polymorphs or degradation of cocrystals upon exposure to aqueous environment, pH or temperature, etc. It is also evident from the literature reports on cocrystal/eutectic/coamorphous/solid solution formulations reviewed so far that most of the solids prepared by cocrystallization have not been studied for their bioavailability, pharmacokinetics and pharmacodynamics. Mere production of cocrystals without investigating its efficacy, pharmacokinetic or pharmacodynamic behavior in biological system would end up in vain. Moreover, preclinical trials followed by clinical trials have needed to develop cocrystals into marketed product.

## Figures and Tables

**Table 1:** Characteristic feature of eutectics, cocrystals and solid solutions.

Property	Eutectic Phase	Cocrystal	Solid Solution	Reference(s)
Structural similarity of the parent molecules	Similar or dissimilar	Similar or dissimilar	Similar	[55]
Isomorphous or non-isomorphous	Isomorphous/non-isomorphous	Isomorphous/non-isomorphous	Isomorphous	[34,55]
Melting point of the solid formed	Lower melting than the parent components	Mostly in between the melting points of parent molecules but may also be higher or lower than the parent molecules	Exhibits a solidus-liquidus melting behavior	[34,55]
Binary phase diagram	‘V’-shaped curve	‘W’-shaped curve	Unary phase diagram	[34,55]
Intermolecular interactions	Short-range and weaker non-covalent adhesive interactions	Stronger and non-covalent adhesive interactions (hydrogen bonding, halogen bonding, Π-Π interactions, etc.)	Stronger and non-covalent cohesive interactions	[34,55]
Arrangement of molecules in crystal lattice	Molecules are randomly arranged	Molecules are well organized and well-packed	Molecules are well organized	[34,55]
Predominant thermodynamic force of the system	Entropy	Enthalpy	Enthalpy	[34,55]
Crystal structure of the solid formed	No significant change from the parent components	Characterized by a new crystal phase formation and hence possess a new crystal structure	Characterized by a new crystal phase formation and therefore possess a new crystal structure	[34,55]
Thermodynamic stability of the solid formed	Less stable	More stable than eutectics	More stable than eutectics	[34]

**Table 2:** Summary of a few literature reports on representative pharmaceutical cocrystals.

Name of the API	Therapeutic Use of the API	Binary/Ternary System	Name of the Coformer	Stoichiometric Ratio of the Cocrystal	Preparation Method	Comments on Dissolution Behavior	Reference(s)
Fluoxetine Hydrochloride	Antidepressant drug	Binary	Benzoic acid,	1:1	Slow evaporation	Fluoxetine HCl-Succinic acid (2:1) and Fluoxetine HCl-Fumaric acid (2:1) cocrystals exhibited enhanced intrinsic dissolution rate than raw Fluoxetine HCl while Fluoxetine HCl-Benzoic acid (1:1) and Fluoxetine HCl-Fumaric acid (2:1) cocrystals showed lower powder dissolution rate than raw Fluoxetine HCl	[57]
			Succinic acid,	2:1			
			Fumaric acid	2:1			
2-[4-(4-chloro-2-fluorophenoxy)phenyl]pyrimidine-4-carboxamide	Sodium channel blocker	Binary	Glutaric acid	1:1	Solution crystallization	The cocrystal phase showed 18 times increased intrinsic dissolution rate than the commercial API	[58]
AMG 517	A transient receptor potential vanilloid 1 antagonist (TRPV1)	Binary	Benzoic acid, *trans*-cinnamic acid, 2,5-dihydroxybenzoic acid, glutaric acid, glycolic acid, t*rans*-2-hexanoic acid, 2-hydroxycaproic acid, l(+)-lactic acid, sorbic acid, l(+)-tartaric acid	1:1	Slow cooling	The cocrystals showed enhanced dissolution in Fasted Simulated Intestinal Fluid (FaSIF) (characterized by bell-shaped profile) than raw AMG 517	[59]
Acyclovir	A guanosine analogue antiviral drug	Binary	Fumaric acid, Glutaric acid	1:1.5, 1:1	Reaction crystallization method	Solubility and permeability of the cocrystals were higher than that of raw acyclovir	[60]
			Tartaric acid	1:1	Solution crystallization and Solvent-drop grinding	Dissolution rate of the cocrystals were faster than anhydrous acyclovir	[61]
Carbamazepine	Anticonvulsant drug	Binary	Salicylic acid	1:1	Slurry method and High-Throughput Screening methods HTS Evaporative experiments, Sonic slurry experiments, Grinding experiments and Reaction crystallization Hot-Melt Extrusion Resonant Acoustic mixing Liquid-assisted grinding, slurry conversion and solution crystallization methods	Not reported	[62,63]
			Maleic acid or maleinic acid,	1:1		Not reported	[64]
			Salicylic acid and few other carboxylic acids	1:1		The extruded cocrystals showed faster dissolution rates than the solvent-crystallized cocrystals and raw carbamazepine	[65]
			Saccharin	1:1			
			4,4′-Bipyridine *p*-aminosalicylic acid	1:1, 2:1:1 cocrystal hydrate and 2:1:1 cocrystal solvate with methanol as solvent		Not reported	[66]
						Carbamazepine- *p*-aminosalicylic acid (1:1) cocrystal showed enhanced dissolution than raw carbamazepine	[67]
Fenofibrate	Helps to lower cholesterol and fat level	Binary	Nicotinamide	1:1	Solution crystallization and Solvent-drop grinding	Cocrystals showed enhanced dissolution than raw nicotinamide	[68]
Agomelatine	Antidepressant	Binary	Urea, glycolic acid, isonicotinamide and methyl-4-hydroxybenzoate	1:1	Agomelatine-urea (1:1) and agomelatine-glycolic acid (1:1) cocrystal was prepared by solution crystallization whereas agomelatine-isonicotinamide (1:1) and agomelatine-methyl-4-hydroxybenzoate (1:1) cocrystal was prepared by melting and recrystallizing in solvent	Cocrystals showed enhanced powder dissolution rate than raw agomelatine	[69]
Benzamide	Neuroleptics and antipsychotics	Binary	Salicylic acid, 3,5-dinitrobenzoic acid, 3-nitrobenzoic acid and 4-hydroxy 3-nitrobenzoic acid	1:1	Solvent-assisted grinding and solvent evaporation	Not reported	[70]
Fluorocytosine	Antitumor agent	Binary	Adipic acid, succinic acid, terephthalic acid, benzoic acid and malic acid	1:1	Solution crystallization	Not reported	[71]
Curcumin	Anticancer, antimalarial and antibacterial compound	Binary	Resorcinol, Pyrogallol	1:1	Solution crystallization and liquid-	The cocrystals exhibited faster dissolution than raw curcumin. Curcumin-pyrogallol cocrystals showed enhanced dissolution than curcumin-resorcinol cocrystal and raw curcumin	[72]
			Phloroglucinol	1:1	assisted grinding	Curcumin-phloroglucinol cocrystal showed lower dissolution than raw curcumin	[73]
			Hydroxyquinol	1:1 and 1:2	Rotary evaporation method Solution crystallization and Solid-state grinding	Curcumin-hydroxyquinol cocrystals showed enhanced dissolution than raw curcumin. Curcumin-hydroxyquinol (1:2) cocrystal exhibited enhanced dissolution than curcumin-hydroxyquinol (1:1) cocrystal and raw curcumin	[24]
			4,4′-Bipyridine-*N*,*N*′-dioxide	1:1	Solution crystallization	Not reported	[74]
Indomethacin	Non-Steroidal Anti-Inflammatory Drug (NSAID)	Binary	Saccharin	1:1	Twin screw extrusion	Indomethacin-saccharin cocrystal showed enhanced dissolution raw indomethacin	[75]

**Table 3:** Summary of few reports on pharmaceutical eutectics available in the literature.

API	Therapeutic Use of API	Coformers Studied	Binary/Ternary System	Mole Fraction of the Coformer	Preparation Method	Comments on Dissolution Behavior	Reference(s)
Curcumin (Form 1)	Anticancer, antimalarial and antibacterial compound	Nicotinamide,	Binary	0.67	Solid-State Grinding	All the eutectics showed enhanced dissolution than raw curcumin	[22]
		Hydroquinone,	Binary	0.5	Solid-State Grinding		[22]
		*p*-hydroxybenzoic acid,	Binary	0.5	Solid-State Grinding		[22]
		Tartaric acid,	Binary	0.5	Solid-State Grinding		[22]
		Ferulic acid,	Binary	0.5	Solid-State Grinding		[22]
		Salicylic acid,	Binary	0.67	Solid-State Grinding		[24]
		Suberic acid	Binary	0.8	Liquid-Assisted Grinding	Not reported	[25]
Pyrazinamide	Anti-tuberculosis drug	Isoniazid,	Binary	0.5	Solid-state grinding	Showed faster dissolution than raw pyrazinamide	[34]
		Isoniazid + Fumaric acid,	Ternary	0.5	Solid-state grinding		
		Isoniazid + Succinic acid,	Ternary	0.5	Solid-state grinding		
Succinamide	Nitrogen supplement in fresh water algae cultivation	Isonicotinamide,	Binary	0.25, 0.5, 0.75	Solid-state grinding	Not reported	[56]
		Isoniazid,	Binary	0.25, 0.5, 0.75	Solid-state grinding	Not reported	
		Fluoxetine HCl	Binary	0.25, 0.5, 0.75	Solid-state grinding	Not reported	
Succinamic acid	Antipruritic and anti-infective agent	Isoniazid,	Binary	0.25, 0.5, 0.75	Solid-state grinding	Not reported	[55]
		Fluoxetine HCl	Binary	0.25, 0.5, 0.75	Solid-state grinding	Not reported	
Hesperetin	Antioxidant, Anticancer and cardioprotective agent	Theophylline,	Binary	0.4	Solvent-drop-assisted solid-state grinding	Hesperetin eutectics showed 2 to 4 times faster dissolution than raw hesperetin	[79]
		Adenine,	Binary	0.67			
		Gallic acid,	Binary	0.6			
		Theobromine	Binary	2:1			
Phenazone	Analgesic, NSAID drug	Phenylbutazone,	Binary	0.550	Not reported	Not reported	[80]
		Phenacetin,	Binary	0.420	Not reported	Not reported	
		Urea	Binary	0.5	Not reported	Not reported	
Sulfadiazine	Antibiotic	Trimethoprim	Binary	0.737	Not reported	Not reported	[80]
Aminophenazone	Analgesic, anti-inflammatory andantipyretic drug	4-aminophenazone,	Binary	0.5	Not reported	Not reported	[80]
		Phenacetin,	Binary	0.320	Not reported	Not reported	
		Phenylbutazone,	Binary	0.5	Not reported	Not reported	
		Phenazone,	Binary	0.463	Not reported	Not reported	
		Etofylline,	Binary	0.142	Not reported	Not reported	
Acetanilide	Analgesic and antipyretic compound	Phenacitin	Binary	0.337	Not reported	Not reported	[80]
Phenacitin	NSAID	Phenobarbital	Binary	0.340	Not reported	Not reported	[80]
Paracetamol	Treatment of pain and fever	Phenobarbital	Binary	0.450	Not reported	Not reported	[80]
Aspirin	Treatment of pain, fever andinflammation	Phenobarbital	Binary	0.327	Not reported	Not reported	[80]
		2-3 parts glycerin or propylene glycol (*w*/*v*)	Binary	Not reported	Proper mixing by melting and followed by allowing to cool to room temperature	Not reported	[78]
		Simvastatin	Binary	66.6% *w*/*w*	Grinding	The eutectic mixture exhibited enhanced dissolution than raw drug	[81]
Sulfanilamide	Antibacterial agent	Phenylbutazone,	Binary	0.867	Not reported	Not reported	[80]
		Benzocaine,		0.867			
		4-aminobenzoic acid		0.404			
Caffeine	CNS Stimulant	Sulfathiazole,	Binary	0.601	Not reported	Not reported	[80]
		Paracetamol		0.619			
Sulfathiazole	Antibiotic	Benzocaine,	Binary	0.936	Not reported	Not reported	[80]
		4-aminobenzoic acid		0.574			
Khellin	Vasodilator	Sulfapyridine,	Binary	0.296	Not reported	Not reported	[80]
		Nicotinic acid		0.194			
2-nitroaniline	-	4-aminobenzoic acid	Binary	0.052	Not reported	Not reported	[80]
Estradiol benzoate	Hormonal therapeutic agent for menopausal symptoms	Estradiol phenyl propionate	Binary	0.837	Not reported	Not reported	[80]
Pyridoxine	Vitamin B_6_	Isoniazid, Nicotinic acid	Binary	0.2, 0.25	Liquid-Assisted Grinding	Not reported	[82]
Irbesartan	Antihypertensive drug	Syringic acid	Binary	50/50% *w*/*w*	Solid-state grinding	Irbesartan eutectic mixtures exhibited 2- to 3-fold enhancement in intrinsic dissolution rate	[83]
		Nicotinic acid	Binary	50/50% *w*/*w*	Solid-state grinding		
		Ascorbic acid	Binary	50/50% *w*/*w*	Solid-state grinding		

**Table 4:** Summary of a few reports available in the literature on drug solid solutions.

Name of the API	Therapeutic Use of API	Binary/Ternary System	Excipient	Reference
Triiodoresorcinol (TIR)	Mycoses treatment	Binary	Triiodophloroglucinol (TIG-O)	[39]
Triiodophenol (TIP)	Disinfectant	Binary	*o*-Triiodoresorcinol (TIR-O)	[39]
Benzoic acid	Antibacterial agent	Binary	4-Fluorobenzoic acid	[38,40]
Isoicotinamide	Treatment of pellagra (caused due to Niacin deficiency)	Ternary	Succinic acid and fumaric acid	[84]

**Table 5:** Summary of a few reports on drug coamorphous solids available in the literature.

API	Therapeutic Use of API	Coformer	Coamorphous Solid Stoichiometry	Preparation Method	Comments on Dissolution Behavior	Reference(s)
Curcumin (Form 1)	Anticancer compound	Artemisinin	1:1	Rotavaporization	The coamorphous solid exhibited 2.6 times faster dissolution than raw curcumin	[28]
		Piperazine	1:2	Ethanol-assisted Grinding	Curcumin-piperazine coamorphous phase showed lower dissolution than raw curcumin at temperature above T_g_ of the coamorphous solid and exhibited higher dissolution than raw curcumin at a temperature below T_g_	[29]
		Folic Acid Dihydrate	1:1	Liquid-Assisted Grinding	The coamorphous phase showed 4 times higher dissolution than raw curcumin	[25]
Indomethacin	Non-steroidal Anti-Inflammatory Drug (NSAID)	Naproxen	1:2, 1:1 and 2:1 (1:1 was the stable form)	Quench cooling	The coamorphous solids exhibited increased intrinsic dissolution rate and 1:1 form showed a synchronized release	[30]
Naproxen	Non-steroidal Anti-Inflammatory Drug (NSAID)	Tryptophan+Proline	1:1:1	Ball milling	Coamorphous phase showed increased intrinsic dissolution rate than the crystalline naproxen	[31]
		Arginine+Proline	1:1:1	Ball milling	Coamorphous phase showed increased intrinsic dissolution rate than the crystalline naproxen	
Atorvastatin calcium	Lipid-lowering agent and in treatment of cardiovascular diseases	Nicotinamide	1:1	Solvent evaporation	Exhibited increased intrinsic dissolution rate than the raw drug	[88]
Sulfathiazole	Short-acting sulfonamide antibiotic	l-Tartaric acid	1:1	Co-milling	Not reported	[32]
		Citric acid	1:1	Co-milling	Not reported	
Ibuprofen	NSAID	Nicotinamide	50% wt./wt.	Loading of mixtures into nanopores of mesoporous silica microspheres	The nanoconfined coamorphous solid showed enhanced dissolution than raw ibuprofen	[33]

**Table 6:** Summary or reports available in the literature on computational screening of coformers.

Computational Screening Technique	Investigated Drug-Coformer Pair	Comments	Reference
Lattice energy prediction	6 cocrystals of caffeine, 8 cocrystals of succinic acid and 12 cocrystals of 4-aminobenzoic acid	Lattice energies of the cocrystals were compared with thesum of lattice energies of individual crystal components	[112]
Lattice energy calculation	Cocrystals of flavonoids	Flexcryst program was used to determine the relative stability of flavonoid cocrystals stored in Cambridge Structural Database (CSD) w.r.t pure crystals and a comparative analysis was made	[113]
Virtual screening tool based on gas phase molecular electrostatic potential surfaces (MEPS)	Diclofenac with 22 coformers,	SSIPs (surface site interaction points) and the interaction site pairing energies of different solid forms (ΔE) determines the stability of the solid forms and thereby enables ranking of Cocrystal Formers based on the calculated probability of cocrystal formation.	[116]
	Piracetam with 29 coformers,		
	Pyrazine carboxamide with 45 coformers,		
	Acetazolamide with 36 coformers,		
	Indomethacin with 57 coformers,		
	a drug candidate with 28 coformers,		
	Furosemide with 28 coformers,		
	Nalidixic acid with 22 coformers and Paracetamol with 37 coformers		
Virtual screening tool based on Functional group interaction energy calculations	Calculations were carried out for Nalidixic acid with 310 coformers	This methodology utilizes surface	[117]
	44 Successful pairs were chosen for experimental studies	site interaction points (SSIPs) calculated from the ab initio MEPS of the isolated molecule in the gas phase	
Virtual screening tool based on gas phase MEPS	Spironolactone and Griseofulvin	Molecular electrostatic potential surfaces enable assession of molecular complementarity between two cocrystal components for determining the cocrystal forming ability	[118]
Virtual screening tool	Several coformers for combination with caffeine ad carbamazepine (identified from literature review)	Interaction site pairing energies of different solid forms were calculated w.r.t pure components and ranking is provided	[126]
Virtual cocrystal screening tool	Isonicotinamide with 97 different coformers	Miscibility affinities of liquid components under supercooledconditions were used as a key parameter to model the cocrystallization propensities	[127]
COSMO-RS (Conductor-like Screening Model for Real Solvents)	Tyrosine kinase inhibitor axitinib, thiophanate-methyl and thiophanate-ethyl benzimidazole fungicides were studied for their solubility behavior using COSMO-RS	Ranking is primarily based on the screening of coformers for API solubility improvement based on calculation of excess enthalpy, *H*ex, between an API-coformer mixture relative to the pure components	[122]
COSMO-RS (Conductor-like Screening Model for Real Solvents)	-	COSMO-RS enables screening of coformers having good solubility with suitable solvents for forming cocrystals	[128]
COSMO-RS	Wide range of pharmaceutical compounds	Virtual coformer screening based on the API-coformer miscibility was performed to screen coformerto produce cocrystals with good Relative Humidity (RH) stability	[123]
Prediction of Hansen Solubility Parameter	Indomethacin with thirty different coformers	Utilization of group contribution method	[121]
Calculation of energy difference	*N*,*N*′-Diphenylureas and Triphenylphosphine Oxide	Relative ΔΔE_int_ between heterodimers and homodimers served as a good predictor of cocrystal formation in the investigated system	[129]
Free energy calculation	Pentoxifylline with coformers, aspirin, salicylic acid, and benzoic acid	Flexcrystprogram was used to calculatethe free energy of experimental and hypothetical crystal structures and then correlated with each other	[130]
In silico screening	Phenylpiperazine derivatives anddicarboxylic acids	Computed values for the mixing enthalpies and solubility advantage factors determine the cocrystallization propensities	[131]
Ab initio screening	Nicotinamide, isonicotinamide, picolinamide and two paracetamol cocrystals were screened for their stability	Lattice energy calculations	[114]
Heat of formation distribution of components	492 pairs	Calculation of Heat of formation (Hf) values	[132]
Fabian approach	974 cocrystal structures were investigated by Fabian approach	Calculation ofQuantitative Structure-Activity Relationship (QSAR) molecular descriptors of cocrystallizing components	[125]
Utilization ofcomputed crystal energy landscapes	Carbamazepine (CBZ)-Nicotinamide (NCT),	Computed crystal energy landscapes of the drug-coformer pairs were compared and analyzed with the binary and ternary phase diagrams	[124]
	Carbamazepine (CBZ)-Isonicotinamide (INA),		
	Carbamazepine (CBZ)-Benzamide (BNZ) and Carbamazepine (CBZ)—Picolinamide (PA)		
Knowledge-based approach	Meloxicam cocrystal and Artemisinin cocrystal (pairs reported in literature were used for the study)	Molecular complementarity, hydrogen bond motifs and multicomponent hydrogenbond propensities were compared and analyzed	[119]
Knowledge-based hydrogen bond prediction	Pyrimethamine drug with carbamazepine, theophylline, aspirin, α-ketoglutaric acid, saccharin, p-coumaric acid, succinimide and L-isoleucine as coformers	Hydrogen bond propensity calculations were carried out to detect the formation of new molecular adducts	[120]
Multistage lattice energy minimization methodology	Cocrystal of 4-aminobenzoic acid with 2,2′-bipyridine and 4-nitrophenylacetic acid as coformers	Involves three important steps: (1) Modelling of intermolecular electrostatic interactions with atomic charges, (2) Interpolation of deformation energies and (3) Enhancement of accuracy	[115]

**Table 7:** Summary of batch processes used for cocrystal synthesis.

Cocrystallization Technique (in Batch Mode)	Principle	Reference(s)
Solid-State Grinding (SSG)/Neat Grinding	Low molecular weight coformer molecules diffuse into the crystal lattice of API molecules, forming intermediate phases such as eutectic or amorphous phase which further lead to a new cocrystal phase	[151]
Liquid-Assisted Grinding (LAG)	Addition of solvent molecules during grinding of API-coformer mixture facilitates diffusion of low molecular weight coformer molecules into the crystal lattice of an API	[151]
Ion and Liquid-Assisted Grinding (ILAG)	Grinding of multiple components along with liquid phase facilitates diffusion of liquid phase into the solid-state thereby facilitating mobility of molecules and exposing the hidden molecules to the surface	[153]
Slow evaporation	Preparing a supersaturated solution and allowing solvent to evaporate slowly at ambient conditions induces primary nucleation, thereby leading to slow growth of crystals	[154]
Ultrasound-assisted cocrystallization	The cavitation energy of ultrasonic waves induces primary nucleation of particles and leads to attainment of supersaturation for cocrystal growth	[155]
Solvent-Mediated Phase Transformation (SMPT)	Keeping the binary mixture of API and coformer into a solvent/solvent mixture for a long-time enables phase transformation to a new phase (cocrystal) or conversion from one polymorphic form to another due to the activity of solvent molecules and their nature of interaction with solute molecules over a period	[163]
Cocrystal formation from moisture	Uptake of moisture by a solid mixture, dissolution of reactant molecules in the mixture, attaining supersaturation leading to cocrystal nucleation and growth are the primary steps involved in cocrystal generation from moisture	[159]
Liquid Anti-Solvent precipitation (LAS)	Supersaturation of molecules (drug + coformer mixture) is attained by mixing of solvent and anti-solvent. This results in increase in supersaturation and nucleation rate and thereby facilitates production of sub-micron cocrystal particles	[161]
Gas Anti-Solvent precipitation (GAS)	In GAS process, gaseous phase is used as anti-solvent instead of a liquid to obtain a cocrystal from a solution of API and coformer from a liquid solution	[162]

**Table 8:** Summary of various reports available in literature on using grinding to synthesize cocrystal.

Type of Grinding	Mechanism of Solid Phase Formation	API	Therapeutic Use of the API	Coformer	Reference(s)
Co-milling	Diffusion of coformer molecules into benzoquinone crystal lattice	Benzoquinone	Antioxidant and anti-inflammatory compound	Diols	[173]
Solid-State Grinding/Neat grinding	Formation of submerged eutectic	Benzophenone	Antioxidant and anti-inflammatory compound	Diphenylamine	[152]
Solid-State Grinding/Neat grinding	Vapor diffusion of naphthalene molecules into the crystal lattice of picric acid	Picric acid	Antiseptic	Naphthalene	[167]
Solid-State Grinding/Neat grinding	Vapor diffusion of naphthalene molecules into the crystal lattice of picric acid	Picric acid	Antiseptic	Naphthalene	[168]
Solid-State Grinding/Neat grinding	Inclusion of water (from the reactant molecules) in the crystal generate a cocrystal hydrate	Theophylline hydrate	Chronic Obstructive Pulmonary Disease (COPD) and Asthma Treatment	Anhydrous citric acid	[151]
Solid-State Grinding/Neat grinding	Inclusion of water (from the reactant molecules) in the crystal lattice to generate a cocrystal hydrate	Anhydrous Theophylline	Chronic Obstructive Pulmonary Disease (COPD) and Asthma Treatment	Citric acid monohydrate	[151]
Solid-State Grinding/Neat grinding	Diffusion of anhydrous citric acid into the crystal lattice of anhydrous theophylline results in anhydrous cocrystal	Anhydrous Theophylline	Chronic Obstructive Pulmonary Disease (COPD) and Asthma Treatment	Anhydrous citric acid	[151]
Solid-State Grinding/Neat grinding	No solid-state reaction	Caffeine	Stimulant	Anhydrous Citric acid	[151]
Solid-State Grinding/Neat grinding	No solid-state reaction	Caffeine	Stimulant	Citric acid monohydrate	[151]
Solid-State Grinding/Neat grinding	The water molecules in the reactant molecule facilitated formation of ‘Caffeine citrate’ cocrystal on grinding	Caffeine hydrate	Stimulant	Anhydrous citric acid	[151]
Solid-State Grinding/Neat grinding	The water molecules in the reactant molecules facilitated formation of ‘Caffeine citrate’ cocrystal on grinding	Caffeine hydrate	Stimulant	Citric acid monohydrate	[151]
Solid-State Grinding/Neat grinding	Solvent vapors of acetone and methanol has catalytic effect on Caffeine-malonic acid cocrystal ground mixture and generates cocrystal	Caffeine	Stimulant	Malonic acid	[174]
Solid-State Grinding/Neat grinding	Inclusion of water (from the reactant molecules) in the crystal lattice to generate a cocrystal hydrate	Theophylline monohydrate	Chronic Obstructive Pulmonary Disease (COPD)and Asthma Treatment	Citric acid monohydrate	[151]
Liquid-assisted grinding	An intermediate amorphous solid phase having high molecular mobility facilitates cocrystallization	Piracetam	Neuroprotective and anticonvulsant drug	Tartaric acid, Citric acid	[171]
Liquid-assisted grinding with water	Inclusion of water as solvent during grinding induces cocrystal hydrate formation	Anhydrous Theophylline	Chronic Obstructive Pulmonary Disease (COPD) and Asthma Treatment	Anhydrous citric acid	[151]
Liquid-assisted grinding with water	Caffeine hydrate acted as an intermediate phase for cocrystal formation	Caffeine	Stimulant	Citric acid	[151]
Liquid-assisted grinding	‘Caffeine citrate’ cocrystal formation	Caffeine	Stimulant	Citric acid	[151]

**Table 9:** Summary of reports available in literature on continuous production of pharmaceutical cocrystals.

Production Method	Principle	API	Therapeutic Use of the API	Coformer	API-Coformer Stoichiometric Ratio	Comments on the Dissolution Behavior	Reference
Twin Screw Extrusion (TSE)/Melt extrusion (ME) processing /Hot-Melt Extrusion (HME)	Raw materials were fed through abarrel containing one or more rotary screws towards a die undercontrolled conditions. The frictional force created between thescrew and the barrel at high temperatures enables good mixing and melting of reactants, reduction in its particle size and thereby result into cocrystals	Indomethacin	Non-Steroidal Anti-Inflammatory Drug (NSAID)	Saccharin	1:1	During powder dissolution studies, 60–70% cocrystals dissolved in first 60 min w.r.t 30% of raw indomethacin	[75]
		Caffeine	Stimulant	Oxalic acid	2:1	Not reported	[183]
		Caffeine	Stimulant	Maleic acid	1:1 and 2:1	Not reported	[185]
		Carbamazepine	Anticonvulsant	Saccharin	1:1	Not reported	[183]
		Nicotinamide	Vitamin of B3	Trans-cinnamic acid	1:1	Not reported	[183]
		Theophylline	COPD and Asthma treatment	Citric acid	1:1	Not reported	[183]
		AMG-517	TRPV antagonist	Sorbic acid	1:1	Not reported	[180]
		Carbamazepine	Anticonvulsant	Trans-cinnamic acid	1:1	The extruded cocrystals exhibited faster dissolution rates than raw carbamazepine and the cocrystals produced by conventional methods	[65]
		Carbamazepine	Anticonvulsant	Saccharin	1:1	TSE processed cocrystals (at temperatures of 120 °C, 135 °C, and 140 °C) at different RPM (5 and 10 RPM) exhibited increased dissolution rates than raw carbamazepine	[65]
		Ibuprofen	NSAID	Isonicotinamide with Xylitol as carrier	1:1	Hot-Melt Extruded cocrystals showed enhanced dissolution rates than raw ibuprofen	[184]
Solid-State Shear Milling (S3M) Technology	The applied shear force in S3M for milling along with the polymeric aid facilitates efficient milling due to generation of high stress fields for grinding	Carbamazepine	Anticonvulsant	Salicylic acid	1:1	Carbamazepine-salicylic acid cocrystals prepared by continuous process exhibited higher dissolution than the cocrystals prepared by batch process. Nearly 95% of drug was dissolved in case of cocrystal samples treated with PolyEthylene Oxide (PEO) and nearly 90% of drug was dissolved in case of cocrystal samples without PEO treatment	[188]
Spray Drying	A technique in which dry cocrystal powders are obtained from a solution or a suspension by evaporating the solvent very rapidly in a fraction of a second by passing a hot air stream	Carbamazepine	Anticonvulsant	Glutaric acid	1:1	Not reported	[186]
		Theophylline	COPD and Asthma treatment	Nicotinamide	1:1	Not reported	[186]
		Urea	Organic carbamide	Succinic acid	1:1	Not reported	[186]
		Caffeine	Stimulant	Glutaric acid	1:1	Not reported	[186]
		Caffeine	Stimulant	Oxalic acid	2:1	Not reported	[186]
		Indomethacin	Non-Steroidal Anti-Inflammatory Drug (NSAID)	Nicotinamide	1:1	Not reported	[186]
Spray Flash evaporation process for preparation of nanococrystals	Subjecting the flashing superheated liquids to a sudden pressure drop followed by atomization in atomization chamber yields nano-sized cocrystals	Caffeine	Stimulant	Oxalic acid	2:1	Not reported	[187]
		Caffeine	Stimulant	Glutaric acid	1:1		
		TNT-20	-	CL20	1:1		
		HMX	-	CL20	1:1		

**Table 10:** Summary of reports on ionic cocrystals available in the literature.

API	Inorganic Ions Used	Counterions Used	Cocrystallization Process	Enhancement in Dissolution Rate	Reference(s)
Nicotinamide (NCT)	CaCl_2_	Nil	Liquid-assisted grinding and slow evaporation in Ethanol	Exhibited lower dissolution than raw NCT	[248]
Piracetam (PRT)	CaCl_2_	Nil	Liquid-assisted grinding and slow evaporation in Ethanol	Exhibited lower dissolution than raw PRT	[248]
Piracetam (PRT)	CaCl_2_	Nil	Liquid-assisted grinding using Methanol and slow evaporation	Not reported	[223]
Barbituric acid (BBA)	KBr	Nil	Kneading, vapor digestion and crystallization in Methanol (MeOH)	Not reported	[250]
Barbituric acid (BBA)	LiBr	Nil	Grinding	Not reported	[250]
Barbituric acid (BBA)	NaBr	Nil	Kneading, vapor digestion and crystallization in MeOH	Not reported	[250]
Barbituric acid (BBA)	RbBr	Nil	Kneading, vapor digestion, crystallization in MeOH, grinding and crystallization in Ethanol (EtOH)	Exhibited higher dissolution than raw BA	[250]
Barbituric acid (BBA)	CsBr	Nil	Grinding and crystallization in EtOH	Exhibited higher dissolution than raw BA	[250]
Barbituric acid (BBA)	CsI	Nil	Grinding and crystallization in EtOH	Exhibited higher dissolution than raw BA	[250]
Brivaracetam (BRV)	MgCl_2_ 6H_2_O and CaCl_2_	Nil	Kneading and crystallization	Not reported	[252]
Seletracetam (SEL)	MgCl_2_ 6H_2_O and CaCl_2_	Nil	Kneading and crystallization	Not reported	[252]
l-Proline (PRO)	Lithium salicylate	Nil	Crystallization in deionized water	Not reported	[253]
l-Proline (PRO)	Lithium hydroxide	Nicotinic acid	Crystallization in deionized water	Not reported	[253]
Piracetam (PIR)	LiCl	Nil	Kneading at different RH conditions and crystallization	There was no significant difference in the Intrinsic Dissolution Rate (IDR) of raw PIR and the ionic cocrystal	[251]
Piracetam (PIR)	LiBr	Nil	Kneading and crystallization	There was no significant difference in the IDR of raw PIR and the ionic cocrystal	[251]
Trimesic acid (H_3_TMA)	2,6-bis(4-pyridylmethylene)cyclohexanone	Nil	Crystallization	Not reported	[254]
Carbamazepine (CBZ)	Sodium iodide (NaI)	Acetyl chloride	Crystallization in methanol	Not reported	[255]
	Sodium iodide (NaI) and Hydrobromic acid (HBr)	Acridinium I_2_X species	Crystallization in methanol	Not reported	[255]
Benzoic acid (BA)	Phenoxy acetic acid	Nil	Slow evaporation technique	Not reported	[249]

**Table 11:** Observations made from a few literature reports on pH-solubility behavior of cocrystals different API-coformer pairs having different ionization properties.

API		Coformer		Solubility Trend w.r.t pH	Reference(s)
Name of the API	Ionizing Nature of API	Name of the Coformer	Ionizing Nature of the Coformer		
Carbamazepine	Non-ionizable	Succinic acid	Diprotic acid	Increase in solubility	[64]
Carbamazepine	Non-ionizable	4-aminobenzoic acid hydrate	Monoprotic acid	Increase in solubility	[63]
Ketoconazole	Weak basic	Adipic acid	Diprotic acid	U-shaped trend in which solubility reached minimum with an increase in pH	[319]
Ketoconazole	Weak basic	Fumaric acid	Diprotic acid	U-shaped trend in which solubility reached minimum with an increase in pH	[319]
Ketoconazole	Weak basic	Succinic acid	Diprotic acid	U-shaped trend in which solubility reached minimum with an increase in pH	[319]
Itraconazole	Basic	l-Tartaric acid	Acidic	U-shaped trend in which solubility reached minimum with an increase in pH (minimum solubilityoccurred in the pH range which is equivalent to the differencebetween two pKa values)	[320]
Gabapentin-lactam	Non-ionizable	Gentisic acid	Acidic	Increase in solubility	[316]
Gabapentin-lactam	Non-ionizable	Benzoic acid	Acidic	Increase in solubility	[316]
Gabapentin-lactam	Non-ionizable	4-aminobenzoic acid	Monoprotic acid	U-shaped trend in which solubility reached minimum with an increase in pH	[316]
Gabapentin-lactam	Non-ionizable	4-hydroxybenzoic acid	Acidic	Increase in solubility	[316]
Gabapentin-lactam	Non-ionizable	Fumaric acid	Diprotic acid	Increase in solubility	[316]
Gabapentin	Zwitterionic	3-hydroxybenzoic acid	Acidic	U-shaped trend leading to increase in solubility	[321]

**Table 12:** Different crystal habits of Sulfadimidine-4-aminosalicylic acid (1:1) cocrystals [305].

S. No	Cocrystal Polymorph	Preparation Method	Crystal Habit	Morphology	Dissolution Level in Deionized Water	Reference
**1**	I	Liquid-assisted co-milling	-	-	Highest	[325]
**2**	II	Solvent evaporation with Ethanol	I	Large prismatic crystals	Higher	
**3**	II	Solvent evaporation with Acetone	II	Large plate-like crystals	Higher	
**4**	II	Solvent evaporation with Ethanol followed by dry milling	III	Small cube-like crystals	Higher	
**5**	II	Spray drying	IV	Microspheres	Lower	

**Table 13:** Summary of a few literature reports on bioavailability of pharmaceutical cocrystals/eutectics/coamorphous solids.

API	Nature of the Solid Phase	Medical Use of the API	Coformer	API-Coformer Stoichiometric Ratio	Cocrystallization Technique	Pharmacokinetic/Pharmacodynamic/Bioavailability Studies	Reference(s)
2-[4-(4-chloro-2-fluorophenoxy)phenyl]pyrimidine-4-carboxamide	Cocrystal	Sodium channel blocker	Glutaric acid	1:1	Solution crystallization	Oral administration of cocrystals and raw drug to dog indicated thatthe cocrystal increased plasma AUC (plasma Area-Under-the-Curve) values by three times than the raw drug	[58]
Curcumin (Form I)	Cocrystal	Anticancer agent	Pyrogallol	1:1	Liquid-assisted grinding	Showed improved pharmacokinetic profile than raw curcumin and did not show toxic effects even at 10 times higher concentrations (at 2000 mg/kg). Curcumin-pyrogallol cocrystal exhibited a bioavailability of 200 mg/kg oral dose in xenograft model	[89]
Curcumin (Form I)	Coamorphous solid	Anticancer agent	Artemisinin	1:1	Rotavaporization	The coamorphous phase exhibited greater dissolution and pharmacokinetic profile than raw curcumin. It also showed higher bioavailability and therapeutic effect than raw curcumin. The coamorphous solid was non-toxic even at a dose of 10 times higher dose at 2000 mg/kg in xenograft model	[89]
Hesperetin	Cocrystal	Antioxidant molecule	Picolinic acid	1:1	Liquid-assisted grinding and solvent evaporation	Showed 20% enhancement in antioxidant activity, 30% hemolysis decrement, 72% inflammation inhibition and exhibited relative bioavailability of 1.36 w.r.t raw hesperetin	[79]
Hesperetin	Cocrystal	Antioxidant molecule	Nicotinamide	1:1	Liquid-assisted grinding and solvent evaporation	Showed 30% enhancement in antioxidant activity, 40% hemolysis decrement, 79% inflammation inhibition and exhibited relative bioavailability of 1.57 w.r.t raw hesperetin	[79]
Hesperetin	Cocrystal	Antioxidant molecule	Caffeine	1:1	Liquid-assisted grinding and solvent evaporation	Showed 50% enhancement in antioxidant activity, 60% hemolysis decrement, 87% inflammation inhibition and exhibited relative bioavailability of 1.60 w.r.t raw hesperetin	[79]
Hesperetin	Eutectic	Antioxidant molecule	Theophylline	1:1.5	Liquid-assisted cogrinding	Showed 30% increment in antioxidant activity w.r.t raw hesperetin and exhibited 2 times greater anti-hemolytic activity than raw hesperetin	[79]
Hesperetin	Eutectic	Antioxidant molecule	Adenine	2:1	Liquid-assisted cogrinding	Showed decreased antioxidant activity w.r.t raw hesperetin and exhibited 1.5 times greater anti-hemolytic activity than raw hesperetin	[79]
Hesperetin	Eutectic	Antioxidant molecule	Gallic acid	1.5:1	Liquid-assisted cogrinding	Showed 50% increment in antioxidant activity w.r.t raw hesperetin and exhibited 2.5 times greater anti-hemolytic activity than raw hesperetin	[79]
Hesperetin	Eutectic	Antioxidant molecule	Theobromine	2:1	Liquid-assisted cogrinding	Showed 30% increment in antioxidant activity w.r.t raw hesperetin and exhibited 2 times greater anti-hemolytic activity than raw hesperetin	[79]
Carbamazepine	Cocrystal	Anticonvulsant	Vanillic acid	1:1	Slow evaporation	Molecular aggregates formed as a result ofthe dissolution of physical mixture reduced the integrity of intestinal cell monolayer ofNCM460 intestinal cells whereas the molecular aggregates which resulted from dissolution of cocrystal phase maintained the integrity of intestinal cell monolayer of NCM460 intestinal cells	[330]
Carbamazepine	Cocrystal	Anticonvulsant	4-Nitropyridine-N-oxide	1:1	Slow evaporation	Molecular aggregates formed as a result of the dissolution of physical mixtureand cocrystal phase maintained the integrity of intestinal cell monolayer ofNCM460 intestinal cells	[330]
Carbamazepine	Cocrystal	Anticonvulsant	Succinic acid	1:1	Slow evaporation	Molecular aggregates formed as a result of the dissolution of physical mixture reduced the integrity of intestinal cell monolayer ofNCM460 intestinal cells whereas the molecular aggregates formed from dissolution of cocrystal phase maintained the integrity of intestinal cell monolayer ofNCM460 intestinal cells	[330]
Dichloroacetic acid	Cocrystal	Anticonvulsant	Cu_2_(valdien)_2_	1:1	Controlled evaporation	Exhibited in vitro cytotoxicity on MCF-7 cancer cell lines	[331]
Quinoxaline	Cocrystal	Anticancer agent	Diacetylmonoxime and 3-thiosemicarbano-butan-2-oneoxime (TSBO)	1:1:1	Slow cooling of boiled solution	The cocrystal phase followed mitochondrial mediated cell death pathway in lung cancer cells, A549 by means of activating caspase 9 and Bax. It also exhibited anticancer activity on breast cancer (MCF-7) cell lines	[332]
Irbesartan	Eutectic	Antioxidant	Syringic acid	1:1	Solid-state grinding	In vivo pharmacokinetic profile of the irbesartan-syringic acid eutectic mixture showed 1.5-fold improvement w.r.t raw irbesartan	[84]
Irbesartan	Eutectic	Antioxidant	Nicotinic acid	1:1	Solid-state grinding	In vivo pharmacokinetic profile of the irbesartan-nicotinic acid eutectic mixture showed 1.6-fold improvement w.r.t raw irbesartan	[84]
Irbesartan	Eutectic	Antioxidant	Ascorbic acid	1:1	Solid-state grinding	In vivo pharmacokinetic profile of the irbesartan-ascorbic acid eutectic mixture showed 2-fold improvement w.r.t raw irbesartan	[84]
Atorvastatin calcium	Coamorphous solid	Lipid-lowering agent, for treatment of cardiovascular diseases	Nicotinamide	1:1	Solvent evaporation	Atorvastatin calcium-nicotinamide coamorphous phase showed improved pharmacokinetic profile in rats than raw atorvastatin calcium	[88]
